# Cell-Derived Nanocarriers of Apoptotic Bodies with an Antimicrobial Peptide for Targeting Intracellular *S. aureus* Infections

**DOI:** 10.1021/acsabm.5c01222

**Published:** 2025-10-22

**Authors:** Valentina Nieto-Marín, Ian Alejandro Fernandez-Soliz, Jorge William Arboleda Valencia, Daniel Pletzer, Danieli Fernanda Buccini, Octávio Luiz Franco

**Affiliations:** † S-Inova Biotech, Programa de Pós-Graduação em Biotecnologia, 186072 Universidade Católica Dom Bosco, Campo Grande, Mato Grosso do Sul 79117-900, Brazil; ‡ Grupo de Investigación FITOBIOL, Instituto de Biología, Facultad de Ciencias Exactas y Naturales, Universidad de Antioquia, Medellín, Antioquia 050010, Colombia; § Laboratorio HERSSEN—Grupo de Investigación en Medicina Integrativa y Fitomedicina, Manizales, Caldas 170002, Colombia; ∥ Centro de Investigaciones en Medio Ambiente y Desarrollo-CIMAD, Universidad de Manizales, Manizales, Caldas 170001, Colombia; ⊥ Department of Microbiology and Immunology, School of Biomedical Sciences, 2495 University of Otago, Dunedin, Otago 9054, New Zealand; # Centro de Análises Proteômicas e Bioquímicas, Programa de Pós-Graduação em Ciências Genômicas e Biotecnologia, Universidade Católica de Brasília, Brasília, Distrito Federal 71966-700, Brazil

**Keywords:** drug delivery, nanotechnology, extracellular vesicles, bacteria, intracellular infections, macrophages

## Abstract

Intracellular infections caused by *Staphylococcus aureus* are challenging due to their ability to evade host defenses, through the development of small colony variants (SCVs) that are resistant to conventional therapies. This study yielded and evaluated size-remodeled apoptotic bodies (ReApoBDs) as cell-derived nanocarriers for the targeted delivery of BotrAMP14, an antimicrobial peptide, to treat *S. aureus*-infected macrophages. ReApoBDs demonstrated high encapsulation efficiencies (∼70%), biocompatibility, sustained drug release over 12 h, colloidal stability, and improved intracellular delivery. The ReApoBDs-BotrAMP14 nanoformulation reduced intracellular bacterial loads while exhibiting lower cytotoxicity compared to the free peptide. Moreover, *in vivo* experiments demonstrated that ReApoBD-BotrAMP14 reduced dermonecrosis by 31.7% and SCV prevalence by 72.9%, more effectively than conventional treatments in a skin abscess model. Finally, the positive correlation between cell viability and bacterial survival highlights the challenge of designing treatments that effectively eliminate intracellular bacteria while preserving host cell integrity. As the first study to develop a ReApoBD-AMP formulation, these findings position ReApoBD-BotrAMP14 as a groundbreaking platform for treating persistent intracellular infections.

## Graphical abstract

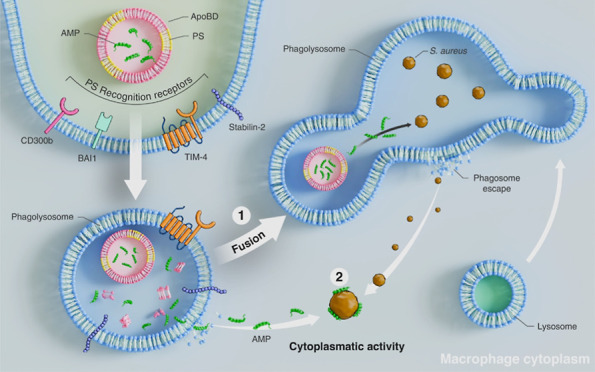

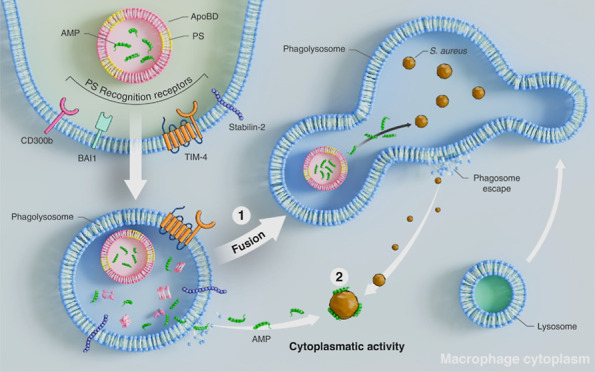

## Introduction

Infectious diseases continue to challenge global health, particularly those caused by pathogens capable of intracellular invasion and persistence. *Staphylococcus aureus* is a common pathogen that causes persistent infections and was associated with more than one million deaths in 2019.^1^ *S. aureus* prominently acts as an intracellular pathogen that often resides within macrophages, evades the host immune response, and complicates treatment, thereby driving resistance to traditional antibiotics.^2,3^ This persistence stems from the pathogen’s ability to transition into small-colony variants (SCVs), a slow-growing, metabolically altered phenotype that enhances survival under hostile conditions.^4,5^

Upon entering macrophages, SCVs encounter a harsh phagolysosome environment, designed to attack and kill pathogens through a cocktail of antimicrobial peptides (AMPs), degradative lysosomal enzymes, reactive oxygen species, and an acidic environment.^6^ However, *S. aureus* SCVs have multiple defense mechanisms that enable them to survive and persist in this hostile environment, expressing superoxide dismutases (e.g., SodA and SodM), catalases (e.g., KatA), and other resistance proteins that allow them to neutralize oxidants and modify their surface to evade immune detection.^7,8^ SCVs pose a significant problem in chronic infections, as they revert to more virulent forms under favorable conditions, causing recurrent infections and treatment failures.^9^ In addition, *S. aureus* SCVs are involved in chronic cases of endocarditis and osteomyelitis, where standard treatments often fail.^10^

Since SCVs are persistent and resistant, innovative therapeutic strategies have been pursued, such as the use of vancomycin, a glycopeptide antibiotic that inhibits bacterial cell wall synthesis and has been a cornerstone in treating MRSA infections.^11,12^ Additionally, ligand-targeted therapies have been developed to facilitate direct intracellular delivery of antimicrobials^13^ and of bioengineered peptides or small molecules, such as NP-6, that disrupt *S. aureus* survival mechanisms^14^ to target and eliminate these variants within macrophages effectively. Researchers have engineered nanoparticle systems to target macrophages selectively by modifying properties such as size, shape, stiffness, and charge, or by incorporating specific surface ligands, including phosphatidylserine (PS) and the macrophage-targeting peptide (M2pep).^13,15^

Moreover, apoptotic bodies (ApoBDs), vesicles generated during apoptosis, represent a promising and unusual avenue for targeted drug delivery. ApoBDs induce chemokine expression to direct macrophages toward phagocytosis^16,17^ and these vesicles also correlate with local anti-inflammatory cytokines’ (e.g., TGF-β and IL-10) expression at infection sites, which favorably modulate immune responses.^18^ Macrophages internalize ApoBDs by recognizing specific “eat me” signals (e.g., PS, ICAM-3, annexin I, and calreticulin) on their surface, while the vesicles suppress “do not eat me” signals (e.g., CD47 and CD31).^19^ More specifically, ApoBD phagocytosis is mediated by redundant pattern recognition receptors (PRRs), most of which directly recognize PS on the vesicle surface.^20^ By mimicking natural apoptotic processes, ApoBDs may provide a biocompatible, efficient means of delivering antimicrobial agents to infected macrophages while reducing immune rejection and systemic toxicity.^21^ Once internalized, effective ApoBD-based antimicrobial strategies rely on the targeted release of encapsulated drugs within phagolysosomes to directly target intracellular *S. aureus* while minimizing cytotoxic effects on the macrophage. These properties make ApoBDs natural and ideal cell-derived nanocarriers for various therapeutic agents, including AMPs, a new and elegant strategy never previously explored.

AMPs are essential to host defense systems and combat diverse pathogens, including bacteria, viruses, and fungi.^22^ Most AMPs disrupt microbial membranes, causing cell lysis and death.^23,24^ Among these, BotrAMP14, an AMP inspired by *Bothrops atrox* venom, demonstrates significant antimicrobial activity against resistant Gram-positive and Gram-negative bacteria, particularly against *S. aureus*.^25^ BotrAMP14’s amphipathic structure appears to enable it to integrate into bacterial membranes, making it a promising candidate for treating infections caused by *S. aureus* that are difficult to treat. However, AMPs face significant challenges in treating intracellular infections due to their limited ability to penetrate host cells and achieve therapeutic concentrations within macrophages.^26^ This limitation underscores the need for advanced delivery systems, such as ApoBDs, which can facilitate targeted AMP delivery to infected macrophages and circumvent SCV-associated resistance mechanisms.

In this study, we pioneered ApoBDs conjugated to BotrAMP14 as a novel strategy to enhance the treatment of intracellular *S. aureus* infections. This approach aims to optimize AMP delivery to infected macrophages, increasing their bioavailability, preventing degradation, and minimizing systemic toxicity.

## Results and Discussion

### Induction of Apoptosis and Isolation of Apoptotic Bodies

Apoptosis was induced in BV-2 (murine microglial) and HeLa (human cervical cancer) cell cultures by combining nutrient starvation and hydrogen peroxide (H_2_O_2_) treatment.^27^ These cell lines were selected due to their distinct membrane charge properties, which influence apoptotic body composition and peptide encapsulation efficiency. Morphological changes indicative of apoptosis, such as membrane blebbing and apoptopodia formation, were observed as early as the third day after treatment. Using Hoechst 33342 dye to stain nuclei, DNA in viable cells produced a uniform blue fluorescence. In contrast, in apoptotic cells, the presence of fragmented DNA and condensed chromatin resulted in altered Hoechst staining patterns, often showing bright, condensed, or fragmented nuclei (Figure 1A). In BV-2 cultures, the accumulation of ApoBDs was observed from day 3, indicating a significant increase in apoptotic cell death (Figure S1). Similarly, HeLa cultures showed a significant increase in ApoBDs by day 4, reflecting the progression of apoptosis induced by the combined treatment method. After successful induction of apoptosis in BV-2 and HeLa cultures, samples were collected when approximately 95% of the cells exhibited an apoptotic morphology. Four samples were collected after sequential centrifugation cycles. The success of the differential centrifugation-based isolation approach was then validated by flow cytometry (FC). Five distinct subpopulations were identified using the annexin V apoptosis marker (A5) and forward (FSC) and side (SSC) scatter: viable cells characterized by SSCintermediate/high, A5low; early apoptotic cells characterized by FSC/SSCintermediate/high, A5low, and FSC/SSCintermediate/high, A5high; necrotic and late apoptotic cells characterized by FSC/SSCintermediate/high, A5high; debris characterized by FSC/SSClow, A5low; and ApoBDs characterized by FSC/SSClow, A5intermediate.

**Figure 1:**
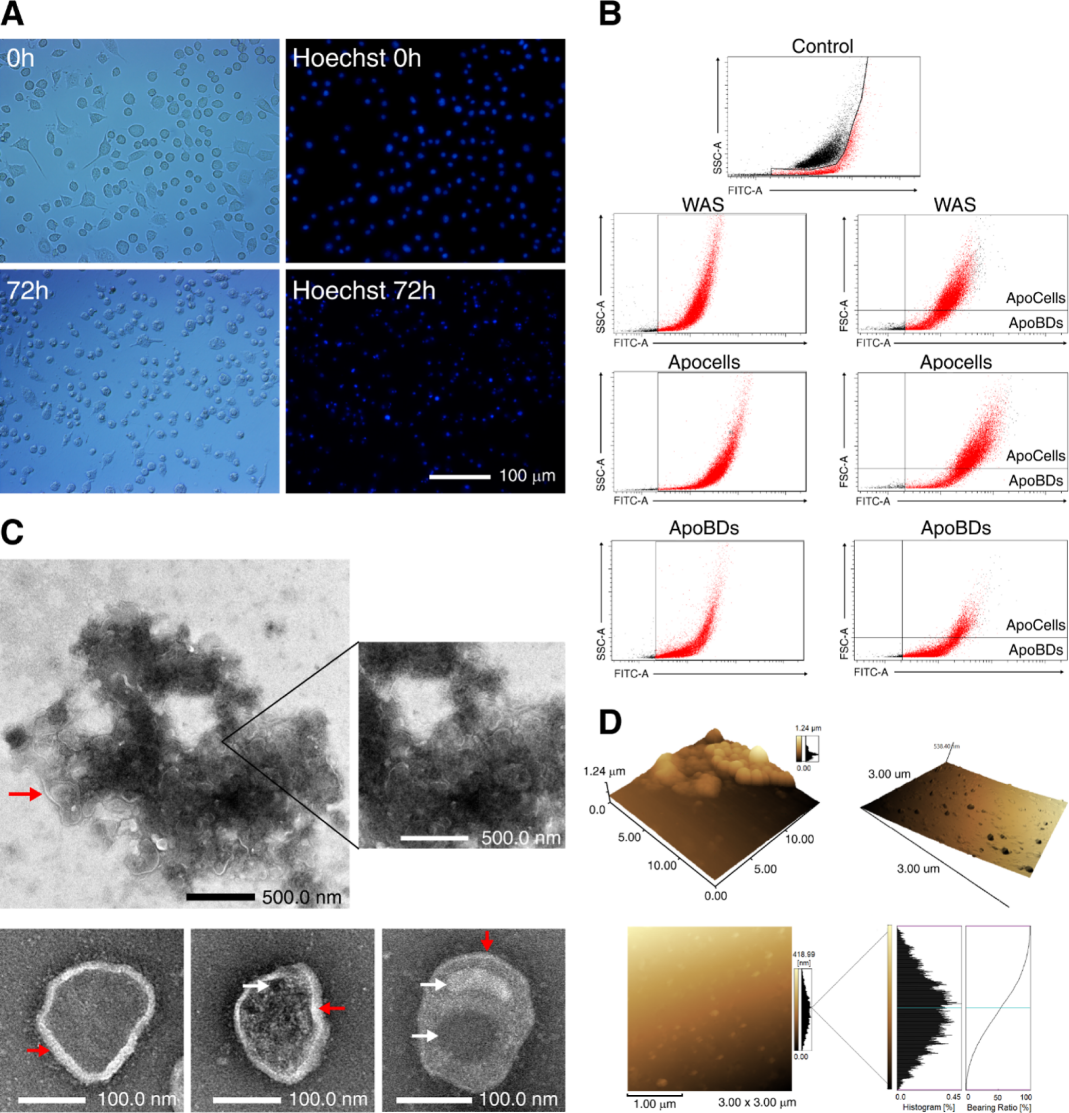
Isolation and observation of apoptotic bodies. (A) Induction of BV-2 apoptosis and staining with Hoechst 33342 (bisbenzimide), a fluorescent nuclear stain to visualize DNA condensation and fragmentation. The figure shows the time-dependent apoptosis process of BV-2 cells as indicated by the fragmented and intensely stained nuclei (10×). (B) Flow cytometry dot plot of each sample. Fluorescein isothiocyanate (FITC)-A/SSC (annexin V/side scatter) dot plot of a control sample containing viable and apoptotic cells (Apocells). FITC-A/SSC and A5/FSC (annexin V/V/forward scatter) were used as a whole apoptotic sample (WAS) control, consisting of a mixture of apoptotic cells and debris, including extracellular vesicles (EVs). FITC-A/SSC and A5/FSC Apocells control with a mixture of apoptotic cells and EVs. FITC-A/SSC and FITC-A/FSC ApoBDs (apoptotic bodies) have a high concentration of these vesicles. (C) Transmission electron microscopy (TEM) micrographs, where negative staining electron microscopy was used to visualize an apoptotic cell (top), with some ApoBDs being formed, and (below) the ReApoBDs (size-remodeled apoptotic bodies). After apoptosis induction, the ApoBDs were collected from BV-2 cultures and used to construct the ReApoBDs. Red arrows indicate the vesicle membrane, and white arrows denote the presence of organelles or cellular debris within the vesicles. (D) Atomic force microscopy (AFM) micrographs of ApoBDs’ and ReApoBDs’ topography.

Figure 1B shows the A5/SSC scatter plot for the control sample gating, where black dot events represent predominantly viable cells with a minor presence of early apoptotic cells (-A5). Red dots indicate a mixture of debris, apoptotic cells, and ApoBDs. Notably, this separation was observed exclusively in the A5/SSC scatter plot for the control treatment, demonstrating distinct subpopulations defined by size, complexity, and apoptotic markers. Figure 1B also shows a mixture of events without clear differentiation in the A5/SSC and A5/FSC scatter plots for the whole apoptotic sample (WAS) gating. This reflects the proportional combination of different subpopulations within the sample, which is particularly evident in the A5/FSC scatterplot, where events representative of apoptotic cells and ApoBDs overlap significantly. In addition, this result also highlights a higher frequency of events associated with apoptotic cells compared to ApoBDs under WAS gating. Conversely, for the ApoBD target sample, a predominant presence of events characteristic of ApoBDs was observed compared to other subpopulations. The strategy of isolating ApoBDs by differential centrifugation successfully isolates vesicles distinct from other cellular subpopulations. This isolation facilitates subsequent characterization and loading steps, which are critical for further experimental analysis. Notably, we established that the flow cytometry-based validation approach does not require secondary markers (e.g., TO-PRO-3) when employed solely for validation purposes rather than for isolation.

### Structural and Morphological Characterization of ApoBDs

Isolated ApoBDs were size-remodeled (ReApoBDs) using an extrusion strategy as described in the methods section, and transmission electron microscopy (TEM) was used to analyze apoptotic cells and ReApoBD samples (Figure 1C). The structure and size of apoptotic cells are clearly defined, with ApoBDs forming at the periphery as part of the apoptotic process. Additionally, ReApoBDs of approximately 100 nm in size, resulting from the extrusion process, can be observed. These have a round shape and contain cellular debris or even whole organelles, a characteristic of this type of extracellular vesicle (EV).^28^ Additionally, atomic force microscopy (AFM) topographic analysis was used to assess the physical structure, integrity, and size distribution of ApoBDs and ReApoBDs. As shown in Figure 1D, ApoBD aggregates appeared round with heights of ∼1.2 μm, consistent with sizes reported in the literature (0.5–5 μm).^17,28^ Similarly, ReApoBDs were analyzed, showing rounded vesicles within a 3 μm × 3 μm area, with a height range of 100–200 nm. However, this range contrasts with the membrane pore used in the extrusion technique (100 nm), which should result in a single population with a diameter of ∼100 nm. The size distribution of the vesicles exhibited a normal distribution, as shown in the histogram (Figure 1D), although the broad curve suggests the possibility of agglomeration. The bearing ratio curve further supports this, indicating homogeneity in size but within the 100–200 nm range. These findings align with issues described in another study, where it was noted that ApoBDs exhibit low stiffness and problematic adhesion, possibly due to their softness and lack of cytoskeleton.^29^ While AFM confirmed the integrity of the vesicles, it does not appear ideal for detailed analysis of the size or shape of ApoBDs.

### Encapsulation Efficiency Determination

After isolating the ApoBDs, the sample was used to prepare formulations using freeze–thaw or extrusion techniques. Vancomycin hydrochloride (VAN), a widely used glycopeptide antibiotic for treating *S. aureus* infections, was included as a control to compare the encapsulation efficiency of a conventional antimicrobial with the bioengineered peptide BotrAMP14. 2-fold dilutions of vancomycin hydrochloride (VAN) and BotrAMP14 (128 μM to 4 μM) were used to create regression eqs (Figure S2A–D). MALDI-ToF analysis was conducted to confirm the presence of VAN and BotrAMP14 (Figure S2E,F). Encapsulation efficiency (EE) was calculated by subtracting the free drug from the total drug mixed with the ApoBDs and verified by quantification of the encapsulated peptide after lysis of the nanoconjugate vesicles. First, the percentage of encapsulated VAN and BotrAMP14 tended to be higher when the extrusion strategy was used, and the vesicle size was remodeled (ReApoBDs) using HeLa and BV-2 ApoBDs (Table S1). For VAN, the results using the HeLa ApoBDs were consistent with another study^12^ where EE between 40% and 60% was achieved using cancer cells as the source of ApoBDs (Figure 2A). Thus, the freeze–thaw method resulted in an EE of 21.5 ± 0.7% for ApoBDs. In comparison, the freeze–thaw method supplemented with extrusion achieved a rate of 45.75 ± 0.63% (Figure S3). In addition to the difference in the encapsulation strategy used, the cell type also made a significant difference, with VAN entrapment tending to be higher in HeLa-derived ReApoBDs compared to BV-2 ReApoBDs. HeLa-derived ReApoBDs exhibited higher VAN EE, potentially due to the elevated cholesterol content in HeLa cell membranes.^30^ Cholesterol-enriched vesicles have been associated with improved vesicle stability and pH gradient maintenance, which has been linked to successful VAN encapsulation.^30,31^ In contrast, for BotrAMP14, a higher percentage of EE (70.8 ± 3.39%) was obtained with extrusion and when BV-2 cells were used as the source of ApoBDs (Figure 2B).

**Figure 2:**
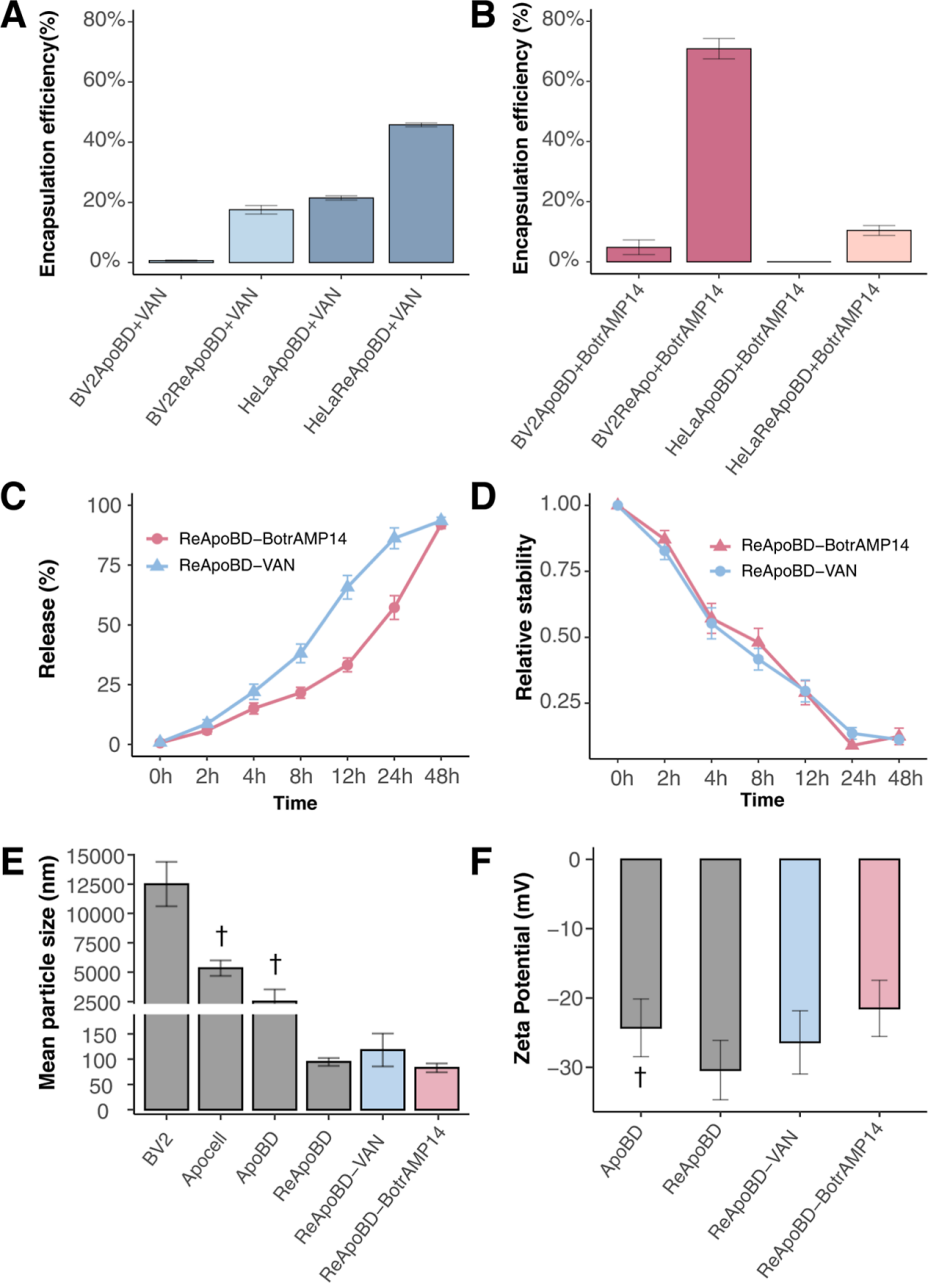
Encapsulation and biophysical evaluation of formulations. Efficiency percentages for different cell types and encapsulation methods. Apoptotic bodies (ApoBDs) or size-remodeled ApoBDs (ReApoBD) from BV-2 (BV-2ApoBD and BV-2ReApoBD, respectively) or HeLa cultures (HeLaApoBD and HeLaReApoBD) were used to load (A) vancomycin (ApoBD/ReApoBD-VAN) and (B) BotrAMP14 (ApoBD/ReApoBD-BotrAMP14). (C) Drug release percentage and (D) relative stability of ReApoBDs formulations after 48 h, and (E) Size frequency distribution of BV-2 vesicles/cell populations, including BV-2 cells (theoretical cell size), apoptotic cells (Apocells), and ReApoBDs formulations with VAN and BotrAMP14. (F) Results of ζ-potential parameters of particles and formulation suspensions from BV-2 culture cells. † Indicates the cell populations that are highly heterogeneous or outside the measurable size range, where only the two most similar technical replicate data were used. Data represent the mean ± SD of three replicates.

BV-2 cells are characterized by a predominantly neutral charge due to their lipid composition.^32^ Their membranes consist mainly of glycerolipids that remain zwitterionic at pH 7. Although ApoBDs are generated after apoptosis, their surface properties still reflect the inherently lower anionic character of the BV-2 parental membrane compared to that of HeLa cells. As a result the membrane charge of BV-2–derived ApoBDs is expected to be less negative than that of HeLa-derived vesicles, with their residual negative character arising primarily from apoptosis-associated events such as phosphatidylserine (PS) exposure and other apoptotic remodeling events (e.g., lipid rearrangements and structural reorganization of membrane components).^32^ In contrast, HeLa cells exhibit a more negatively charged outer leaflet membrane, resulting from O-glycosylation alterations in the membrane glycoproteins.^33^ These modifications differ from those in noncancerous cervical epithelial cells.^33,34^ This increased negative charge in HeLa membranes likely causes ApoBDs from HeLa cells to exhibit stronger electrostatic interactions with the cationic BotrAMP14 peptide, leading to vesicle destabilization and reduced EE. Such electrostatic interactions are also influenced by the surrounding aqueous environment, where factors like pH and ionic strength can modulate the balance between peptide binding, membrane stability, and encapsulation efficiency.^30–32^

In contrast, cationic peptide encapsulation in vesicles composed of neutral (zwitterionic) phospholipids, such as phosphatidylcholine (PC), is more stable because the interactions with the peptide are less likely to disrupt the membrane.^35–38^ This stability is supported, for example, by studies demonstrating higher EE of a cationic AMP (nissin) in neutral lipid liposomes, such as PC, compared to those made with anionic phospholipids like phosphatidylglycerol (PG).^36–38^ Nisin EE was highest (34.6%) when using liposomes containing 85% neutral PC and only 1% negatively charged phosphatidylinositol (PI), demonstrating the positive impact of neutral lipids on EE.^36–38^

This finding aligns with our data, which shows a higher EE of BotrAMP14 in BV-2-derived ApoBDs. The lower negative charge and more stable membrane composition of BV-2-derived ApoBDs may contribute to their superior ability to encapsulate BotrAMP14. Additionally, reduced PS exposure in BV-2-derived ApoBDs may minimize nonspecific interactions, further enhancing peptide conjugation and intracellular bioavailability, consistent with descriptions from prior studies on biogenic vesicles.^17,39^

### Drug Release and Stability

The drug release and stability profiles of the nanoformulations (ReApoBD-VAN and ReApoBD-BotrAMP14) were evaluated over a 48 h period. Drug release and stability were measured at 0, 2, 4, 8, 12, 24, and 48 h (Figure 2C,D). Both formulations showed a clear trend of increasing release over time. For ReApoBD-VAN, the release was minimal at 0 h but increased at 2 h (from 5.76% to 11.76%), reaching almost complete release (from 91.69% to 96.46%) by 48 h. Similarly, ReApoBD-BotrAMP14 had a minimal release at 0 h, increasing to 3.58% to 8.58% at 2 h and reaching 88.96% to 94.83% at 48 h. Stability decreased as drug release progressed. For ReApoBD-VAN, stability dropped from 1 at baseline to 0.3 by 12 h and 0.1 by 48 h. ReApoBD-BotrAMP14 exhibited a similar pattern, with stability decreasing from 1 at baseline to 0.5 at 4 h and 0.09 at 48 h. This decline suggests a loss of colloidal stability, likely due to weakened electrostatic repulsion between particles, promoting aggregation or structural rearrangements in the ReApoBDs as the drug was released.^40^

To further examine the role of acidic and reductive environments, release and stability were also tested in a lysosomal-mimicking buffer (pH 5.0 supplemented with 10 mM GSH). In these experiments, the amount of free BotrAMP14 present in the supernatant at each time point. Under lysosomal-mimicking conditions, the detected free peptide fraction increased rapidly, with ∼50% of the peptide detected between 4–8 h and near-complete release by 24 h (Figure S4A). This accelerated release contrasts with the more gradual profile observed in PBS, indicating that destabilization of the ReApoBD carrier in acidic/reductive conditions is a key driver of peptide exposure. Importantly, the correlation between ζ-potential decay and peptide release was also evident in this buffer (Figure S4B), supporting that surface charge neutralization and colloidal destabilization are principal drivers of payload release under stress conditions.

Mechanistically, three factors likely contribute to these observations:^41^ (i) acidification (pH 5) increases protonation of ionizable groups in the vesicle membrane and reduces ζ potential, thereby decreasing electrostatic repulsion and favoring aggregation or structural rearrangement; (ii) 10 mM GSH, used here to mimic a strong intracellular reducing environment, may perturb protein–lipid interactions or redox-sensitive components, promoting vesicle destabilization; and (iii) BotrAMP14, being highly cationic and amphipathic, can interact strongly with anionic membrane components (e.g., phosphatidylserine), neutralizing surface charge and directly perturbing membranes. The combination of these effects explains the parallel decline in measured stability and the accelerated peptide release under lysosomal-mimicking conditions.^41^

Furthermore, nanoformulations freeze–thaw stability was evaluated under frozen conditions (−80 °C in PBS). Results indicated a marked decline in colloidal stability over time, with relative stability values decreasing by approximately 50% after 3 days and reaching nearly zero by day 5 (Figure S5). These findings, along with the sustained release and short-term colloidal stability results, suggest that the remodeling strategy used to generate ReApoBDs contributed to improved vesicle stability under both suspension and storage conditions. While ApoBDs have been reported to exhibit short-lived stability under physiological conditions (∼3–6 h at 37 °C),^32^ the structural modifications introduced during ReApoBD generation may offer advantages in preserving integrity during formulation handling and storage.

### Biophysical Characterization of the Particles

After extrusion, DLS and zeta potential analyses were performed to determine the hydrodynamic diameter (d­(H)), ζ-potential, and stability of the vesicles and other cell populations in suspension. The DLS measurements provided a correlation curve that served as quality control for particle movement in suspension (Figure S6A). Figure 2E shows the particle size distribution based on their d­(H). In our study, the most reliable measurements were obtained for ReApoBDs and their drug-loaded formulations, which consistently exhibited a uniform size of around 100 nm, confirming successful size control through extrusion. Moreover, the ReApoBD-VAN and ReApoBD-BotrAMP14 nanoformulations showed d­(H)­s values slightly higher and lower than those of the empty ReApoBDs, respectively. Additionally, the polydispersity index (PDI) values, below 0.7, confirmed that the samples were monodisperse and suitable for DLS analysis (Table S1).^42^

In contrast, ApoBD vesicles exhibited broad size distributions, ranging from 1000 to 5000 nm (Figure S7), reflecting their physiological heterogeneity.^28^ These measurements are less reliable for quantitative interpretation, as ApoBDs constitute a polydisperse population with diameters ranging from 0.5 to 5 μm and variable structural integrity. Therefore, DLS data from ApoBDs should be considered only descriptive, and not directly comparable to those of ReApoBDs.

Zeta potential analysis further validated sample quality through a V-shaped phase diagram (Figure S6B), which reflects the relationship between the particle’s surface charge and the ionic strength of the surrounding liquid. For ReApoBDs and their formulations, the ζ-potential values provided robust information on colloidal stability and electrokinetic properties. A value greater than ±30 mV generally indicates stability due to electrostatic repulsion, preventing aggregation.^43^ Pure ApoBDs exhibited mean values ranging from −27.1 mV to −23.4 mV (Table S1), but given their broad size range and heterogeneity, these results cannot be interpreted with the same confidence as those from ReApoBDs. In contrast, ReApoBDs showed more negative values (−36.16 mV to −31.45 mV), which is consistent with their smaller and more uniform size after extrusion. This difference in zeta potential values is likely because smaller particles often exhibit higher absolute values due to their increased surface area-to-volume ratio, which may enhance the exposure of ionizable surface groups. This can result in greater surface charge density, especially under constant medium conditions.^44^ The negative zeta potential values of both ApoBD and ReApoBD populations are attributed to the presence of negatively charged molecules, such as PS, on their surface, which facilitates macrophage interaction and uptake.^45–47^ For ReApoBD-VAN, the zeta potential mean values ranged from −28 mV to −25.56 mV (Table S1), which is consistent with previous reports on similar ReApoBD or liposomal VAN formulations.^12,48,49^ These slightly less negative values, compared to empty ReApoBDs, may result from an increase in formulation size, partial shielding of surface charges, or the incorporation of positively charged molecules. Despite this reduction, moderate colloidal stability is maintained. ReApoBD-BotrAMP14 exhibited zeta potential values from −17.3 mV to −21.4 mV, representing the most significant reduction in negative charge. However, the absence of a shift to positive values suggests encapsulation rather than surface adherence. Given that BotrAMP14 is cationic, it likely introduces positive charges that neutralize some surface negativity, leading to a less negative zeta potential. This was further supported by measurements of empty ReApoBDs mixed with the peptide in suspension, which exhibited near-zero values (4.98 ± 4.63 mV), suggesting the presence of positive charges. This phenomenon has also been reported in studies on bacterial and liposomal suspensions treated with cationic peptides^50^ and peptide antibiotic NK-2.^51^

### Cytotoxicity Evaluation of the Formulations

The cytotoxicity of the ReApoBD-VAN and ReApoBD-BotrAMP14 nanoformulations was assessed using the MTT assay to measure cell viability. Results for untreated RAW cells were compared with free drugs (VAN and BotrAMP14) and their nanoformulations using ApoBDs derived from BV-2 cells (Figure 3A). Empty BV-2-ReApoBDs exhibited low cytotoxicity, with cell viability of approximately 90%, indicating biocompatibility and suitability as drug carriers. For VAN, both free and formulated drugs showed dose-dependent cytotoxicity, with the ReApoBD-VAN exhibiting slightly lower cytotoxicity at higher concentrations (24.6% to 20.6%) compared to free VAN (27.8% to 22%). For BotrAMP14, the free peptide exhibited dose-dependent cytotoxicity, with lower concentrations associated with higher cell viability (ranging from 45.35 ± 2.17 at 256 μM to 89.93 ± 2.09 at 2 μM). Similarly, the ReApoBD-BotrAMP14 formulation showed reduced cytotoxicity at decreasing concentrations (40.65 ± 2.32 at 256 μM to 79.43 ± 2.12 at 2 μM). The formulation of VAN and BotrAMP14 was associated with a slightly reduced cytotoxicity at higher concentrations, likely due to the controlled release mechanism of the vesicles over 12 h (Figure 2C), as described in previous studies.^52–54^ Although both free and formulated versions of VAN and BotrAMP14 exhibited dose-dependent effects on RAW cell viability, no statistically significant differences were observed between the treatments at any concentration.

**Figure 3:**
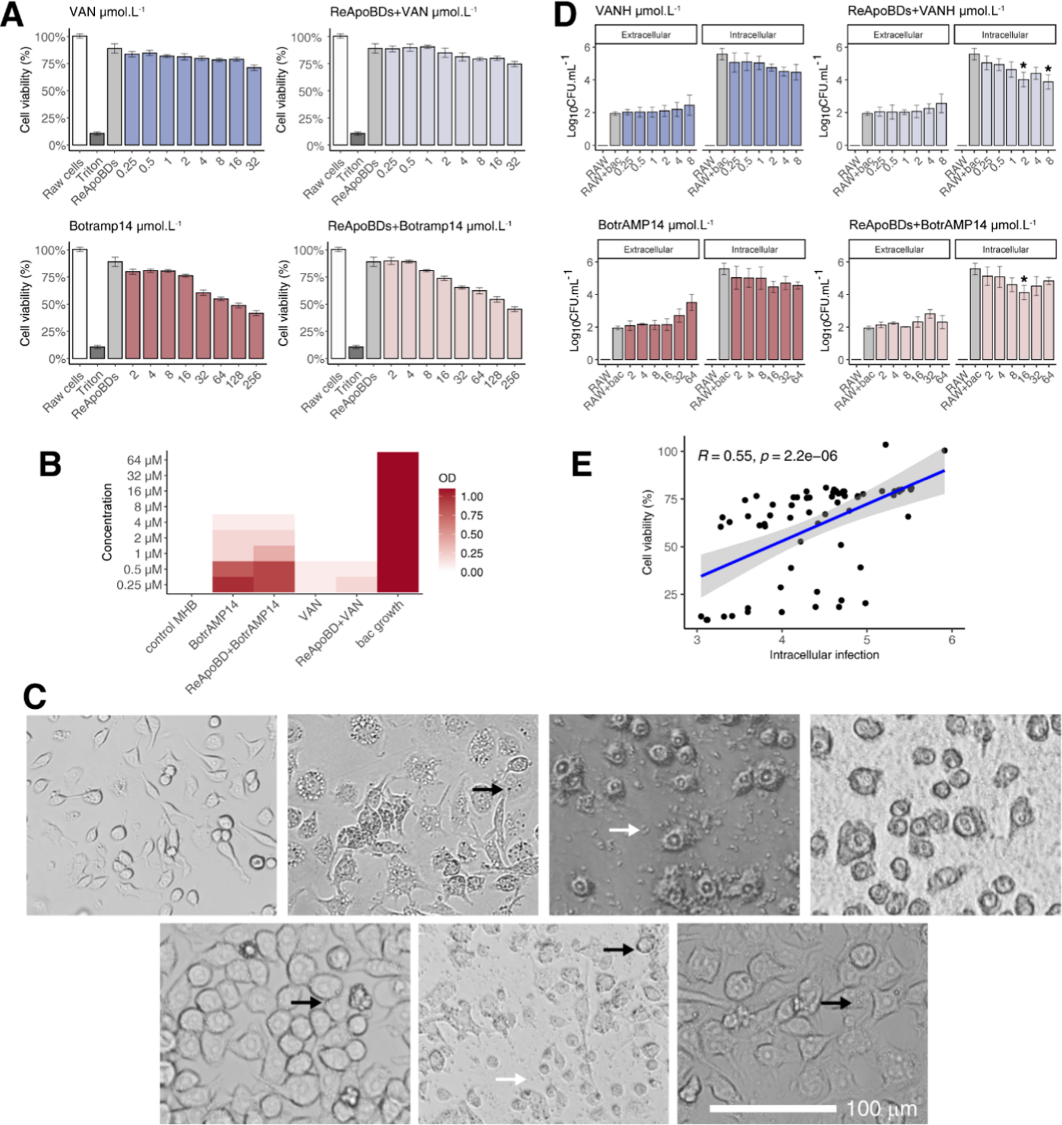
Evaluation of the biological efficacy of the formulations. (A) Cytotoxicity of free compounds, formulations, and ReApoBDs (size-remodeled apoptotic bodies) against RAW 264.7 macrophage cells. (B) Minimum inhibitory concentration (MIC) assay to evaluate the antibacterial activity of vancomycin (VAN, BotrAMP14, and their formulations against the *S. aureus* strain. (C) Images of intracellular bacterial killing assays in macrophages. (first, top) RAW cell morphology in cultures without *S. aureus* and coculture of (second right, top) RAW cells with *S. aureus* Aurora after 24 h. Infected RAW cells were treated after 24 h with (third right, top) ReApoBDs 78 μg mL^–1^, (last, top) VAN 256 μM, and (first, bottom) BotrAMP14 256 μM or (middle, bottom) ReApoBD-VAN 8 μM and (last bottom) and ReApoBDs-BotrAMP14 16 μM. Cultures were observed at 40× magnification using an inverted microscope. The black arrow indicates the presence of bacteria in the intracellular space, and the blue arrow indicates the presence of bacteria in the extracellular space. (D) Quantifying bacterial infection in a coculture of RAW cells with *S. aureus* Aurora after 24 h of treatment, expressed as the Log10 of CFU recovered from the extracellular and intracellular spaces. (E) Pearson correlation between the cytotoxicity of the treatments on RAW cells and the quantification of intracellular *S. aureus* infection (*p*-value indicates the result of a Student’s *t*-test). * Indicates significant effects (*p*-values <0.05) from ANCOVA, using macrophage viability as a covariate. All data are median ±SD of three replicates.

Designing antimicrobial treatments for intracellular delivery necessitates a careful balance in drug efficacy while minimizing cytotoxicity to host cells. The observed reduction in cytotoxicity upon formulation was similar to another nanostructured antimicrobial approach, such as the self-assembled peptides F3FT and N3FT, which demonstrated potent intracellular antibacterial activity with low cytotoxicity in RAW 264.7 cells.^55^ These peptides efficiently penetrated macrophages and eliminated intracellular *S. aureus* while maintaining IC_50_ values above 64 μM, in contrast to melittin, which exhibited high toxicity at just 4 μM.^55^ These findings highlight the potential of engineered nanosystems to enhance the therapeutic index by optimizing intracellular drug delivery and cellular interactions.

### *In Vitro* Antibacterial Evaluation

The susceptibility of the *S. aureus* Aurora strain to VAN and BotrAMP14 and ReApoBD-VAN and ReApoBD-BotrAMP14 nanoformulations was evaluated *in vitro* using planktonic and macrophage infection models. MIC tests confirmed the susceptibility of the Aurora strain to VAN (MIC = 0.5 μM) based on CLSI standards (≤1.4 μM). BotrAMP14, in contrast, exhibited an MIC of 4 μM. In addition, the ReApoBD-VAN and ReApoBD-BotrAMP14 nanoformulations also exhibited MIC values similar to the free drug, indicating the successful release of the drug during the first 24 h (Figure 3B, Table S2).

In the *in vitro* macrophage infection model, intracellular bacterial loads in untreated cells reached 5.7 ± 0.3 Log10CFU, while extracellular levels stabilized around 2 ± 0.1 Log10CFU. Free VAN reduced intracellular loads to an average of 4.4 ± 0.5 Log10 CFU with a maximum reduction of 1.7 Log10CFU at the highest concentration (8 μM) (Figure 3C,D). The ReApoBD-VAN nanoformulation at the same concentration further reduced intracellular bacterial loads to an average of 3.9 ± 0.4 Log10CFU, with reductions of up to 2.2 Log10CFU observed, underscoring its enhanced efficacy in penetrating macrophages. These findings are consistent with reconstructed ApoBDs loaded with VAN, which achieved a two-Log10 reduction in intracellular bacterial loads within RAW 264.7 cells.^12^ Similarly, free BotrAMP14 achieved intracellular decreases to an average of 4.4 ± 0.3 Log10CFU at 16 μM, resulting in efficacy comparable to VAN (Figure 3C,D). Thus, the ReApoBD-BotrAMP14 nanoformulation showed superior activity, achieving an average of 4.1 ± 0.3 Log10CFU at 16 μM, with reductions extending to 2.1 Log10CFU, demonstrating the advantages of encapsulation for intracellular delivery.

These findings align with recent research on antimicrobial nanoformulations for intracellular *S. aureus* eradication.^55^ An study developed self-assembling nanopeptides (F3FT and N3FT) with dual antibacterial and cell-penetrating properties.^55^ That study demonstrated that these nanopeptides eliminated up to 98.3% of intracellular *S. aureus* in RAW 264.7 cells at 32 μM, significantly outperforming vancomycin, which achieved only 32.6% reduction at the same concentration. The higher efficacy of F3FT and N3FT was attributed to their efficient cell penetration and their dual mechanism of membrane disruption and ROS accumulation. While our ReApoBD-VAN and ReApoBD-BotrAMP14 nanoformulations also improved intracellular bacterial clearance, their effectiveness remains dependent on antibiotic release and macrophage uptake, highlighting a complementary but distinct strategy compared to self-assembled nanopeptides.

Notably, our study quantified extracellular bacterial loads (intracellular bacteria released after the total elimination of bacteria that did not achieve intracellular infection) without using antibiotics in the medium. This distinguishes intracellular bacterial reductions caused by antibacterial activity and cytotoxic effects. Thus, for extracellular bacteria, any treatment significantly reduced bacterial loads, which remained stable at ∼2 Log10CFU. Pearson correlation analysis (Figure 3E) revealed a moderate positive correlation (*R* = 0.55, *p*-value = 2.2 × 10^–6^) between macrophage viability and intracellular bacterial loads. This suggests that treatments with greater cytotoxic effects tended to exhibit increased bacterial clearance. However, this observation highlights the need to balance antimicrobial efficacy with cytocompatibility, rather than attributing bacterial killing solely to cytotoxic effects. ReApoBD-VAN and ReApoBD-BotrAMP14 nanoformulations that achieved a higher antimicrobial-to-cytotoxicity ratio yielded more favorable outcomes (Figure S8).

### *In Vivo* Antimicrobial Efficacy of the ReApoBD-BotrAMP14 Nanoformulation

The antimicrobial efficacy of the ReApoBD-BotrAMP14 nanoformulation was evaluated using a subcutaneous abscess model in mice infected with the *S. aureus* Aurora strain (Figure 4A). The ReApoBD-BotrAMP14 nanoformulation treatment significantly reduced abscess size by 31.7%, with an average area of 43.59 mm^2^ compared to 63.83 mm^2^ in the saline-treated group (Figure 4B,C). Although variability was observed across individual animals, this was expected due to the inclusion of both male and female mice, which is known to influence lesion progression during *S. aureus* skin infections through sex-dependent immune responses—such as estrogen-mediated modulation leading to reduced dermonecrosis in females.^56^ The *in vivo* model proved to be well-suited to this study, as it induced the appearance of SVC phenotypes. The identification of SCVs was based on direct observation of colony morphology on Mueller–Hinton agar. SCVs were distinguished from normal *S. aureus* colonies by their significantly smaller size, reduced pigmentation (appearing pale or translucent instead of golden or yellow-cream), rough and dry texture, and prolonged incubation time required for visible growth (>24 h compared to 18–24 h for normal colonies). These characteristics are consistent with previously described SCV phenotypes in the literature.^9,57^ The ability to detect and reduce SCVs in this model underlines the ReApoBD-BotrAMP14’s potential to target these difficult-to-eradicate bacterial subpopulations. Our results showed that the ReApoBD-BotrAMP14 treatment effectively reduced SCV prevalence, with treated animals exhibiting a relative frequency of 0.271 compared to the control (Figure 4D). This finding suggests a possible intracellular effect of the treatment, addressing a key limitation of conventional strategies. Additionally, in the total bacterial load analysis, the treated groups showed a reduction of 0.97 Log10 CFU ± 0.37 per abscess compared to the control (Figure 4E).

**Figure 4:**
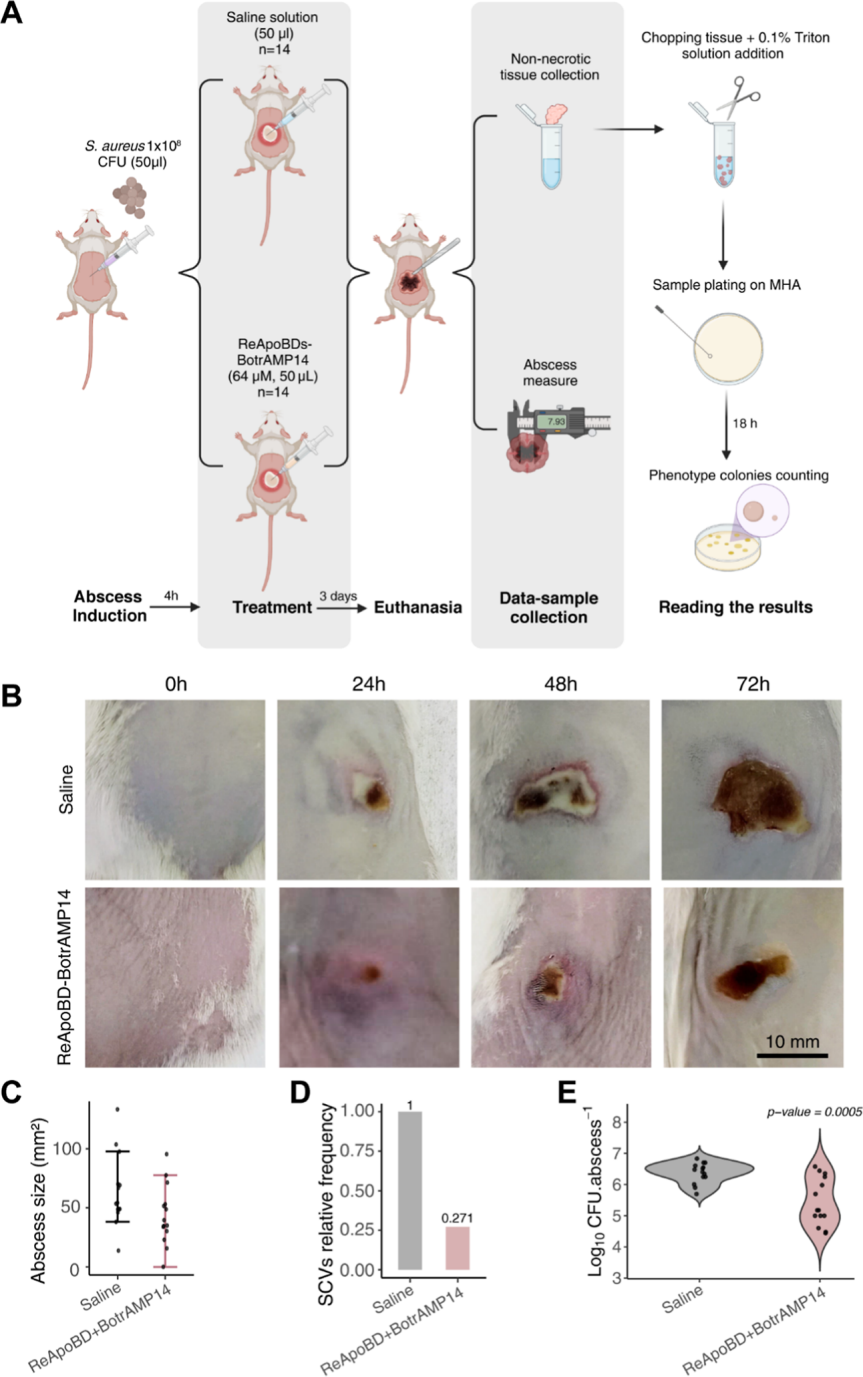
*In vivo* evaluation of ReApoBD-BotrAMP14 nanoformulation. (A) Experimental design outlining the timeline of mouse treatment conditions, including the use of the *S. aureus* Aurora strain to induce abscess formation, peptide nanoformulation treatments, and sample collection. Created in BioRender. (B) Images of Aurora *S. aureus* cutaneous infection on Swiss Webster mice over 3 days. (C) Abscess area expressed in mm^2^ for saline and nanoformulation-treated groups. (D) Relative frequency of presence of *S. aureus* Aurora small colony variants (SCVs) in Mueller–Hinton agar (MHA) plaques of plated samples collected from non-necrotic abscess tissue of animals treated with the peptide nanoformulation compared to the saline-treated group. (E) Effect of ReApoBD-BotrAMP14 nanoformulation on total bacterial concentration recovered from non-necrotic tissue of *S. aureus*-induced abscess. The *p*-value indicates the result of a Student’s *t*-test. All data represent three biological replicates with at least 4–5 mice group.

Previous studies have highlighted the difficulty of eradicating *S. aureus* SCVs, particularly in models of chronic infection. For instance, in a rabbit model of chronic osteomyelitis, vancomycin-hydroxyapatite-loaded cements effectively targeted SCVs but required prolonged administration (42 days) and extremely high doses (80,000–240,000 mg kg^–1^) for complete eradication.^58^ Similarly, in the mouse peritonitis model, intracellular-active antibiotics such as linezolid (17 mg kg^–1^) and dicloxacillin (60 mg kg^–1^) controlled intra- and extracellular infections but failed to eliminate SCVs after a single dose.^59^ In a mouse mastitis model, cefapirin (10–25 mg kg^–1^) showed limited efficacy against hemin-dependent SCVs,^60^ while combination therapies like gentamicin and β-lactams effectively eliminated normal *S. aureus* phenotypes but remained ineffective against SCVs.^61^

In contrast, our study demonstrated that a single subcutaneous dose of ReApoBD-BotrAMP14 (0.3 mg kg^–1^) significantly reduced SCV prevalence compared to the control, achieving a notable effect with a markedly lower dose than those reported in the aforementioned studies using conventional antibiotics, and without the need for prolonged treatment. This result highlights the potential of ReApoBD-BotrAMP14 as a highly effective and less intensive strategy for targeting SCVs in persistent *S. aureus* infections. Similar results were observed with the self-assembled peptides F3FT and N3FT, which showed high antimicrobial efficacy in a mouse model of *S. aureus* peritonitis-sepsis.^55^ A single intraperitoneal injection of F3FT or N3FT (10 mg.kg^–1^) showed greater efficacy in eliminating intracellular bacteria in peritoneal macrophages compared to VAN, probably due to their better cellular penetration. These findings support the efficacy of nanoscale antimicrobial strategies in controlling intracellular *S. aureus* infections, offering a promising alternative to conventional antibiotics that often fail to eradicate SCVs and persistent bacterial populations. These findings support the hypothesis that nanoscale antimicrobial strategies may contribute to controlling intracellular *S. aureus* infections, offering a promising alternative to conventional antibiotics that often fail to eradicate SCVs and persistent bacterial populations.

Taken together, these results support the hypothesis that ReApoBD-BotrAMP14 may act on intracellular reservoirs of bacteria while also controlling extracellular growth, reinforcing its potential as a promising therapeutic strategy for persistent *S. aureus* infections. The observed impact on SCVs and total bacterial burden suggests its ability to address the complexity of targeting both intracellular and extracellular compartments.

Our findings align with those reported in another study.^12^ It demonstrated that reconstructed apoptotic bodies (ReApoBDs) preferentially accumulate in macrophage-rich organs (i.e., liver and spleen), are efficiently internalized by macrophages, and significantly enhance the intracellular antibacterial activity of formulated drugs compared to the free compound. These mechanistic insights support our interpretation that the reduction of SCVs observed in our model may derive from the intracellular activity of the ReApoBD-BotrAMP14 formulation, facilitated by vesicle uptake through apoptotic “eat-me” signals and sustained drug release. This interpretation is further supported by a study^62^ in which menD and hemB SCV mutants displayed comparable infectivity to their parental strain in a rabbit endocarditis model, but responded differently to oxacillin therapy: while bacterial densities of the hemB mutant and the parental strain were significantly reduced across tissues, the menD mutant persisted in kidney (*p*-value = 0.17) and spleen (*p*-value = 0.39) despite treatment. These findings highlight that SCVs can colonize multiple organs and, depending on their auxotrophy, maintain tissue-specific persistence under antibiotic pressure, underscoring the need for therapeutic strategies that effectively target these recalcitrant subpopulations.

### Intracellular Uptake Assay Using Confocal Microscopy

Confocal microscopy was used to evaluate the intracellular uptake of ReApoBDs-BotrAMP14 and its interaction with *S. aureus* in RAW 264.7 macrophages (Figure 5A,B). Three experimental conditions were designed to compare the intracellular behavior of free BotrAMP14 and ReApoBD-BotrAMP14 treatments, with a focus on uptake dynamics and localization at varying incubation times. First, RAW cells were incubated with free BotrAMP14 for 20 min. The absence of 5-TAMRA fluorescence indicated that the free peptide could not penetrate the cellular membrane or localize in intracellular compartments, highlighting the peptide’s limited capacity for intracellular delivery (Figures 5A, S9A).

**Figure 5:**
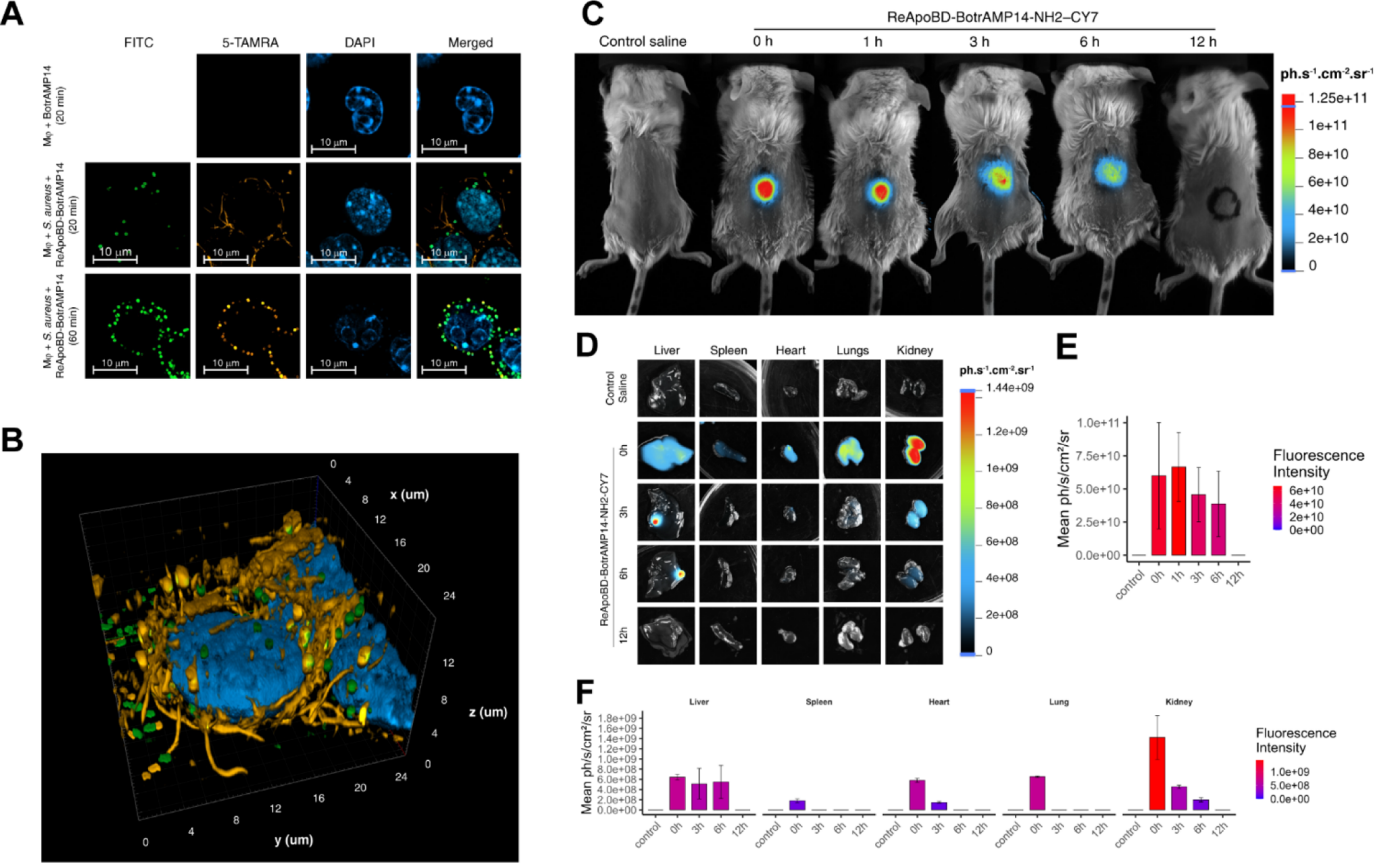
Intracellular uptake and *in vivo* 2D biodistribution of the ReApoBD-BotrAMP14 nanoformulation. (A) Confocal microscopy images showing fluorescence channels for FITC (*S. aureus*, green), 5-TAMRA (free and nanoformulated peptide, orange), DAPI (nuclei, blue), and merged images. Treatments include free peptide and ReApoBD-BotrAMP14 at different incubation times. (B) Volumetric rendering of fluorescence signals from confocal microscopy, providing a three-dimensional view of the signal distribution of the 20 min ReApoBD-BotrAMP14 treatment. (C) *In vivo* 2D fluorescence imaging of BALB/c mice treated with the ReApoBD-BotrAMP14 nanoformulation, showing fluorescence at the injection site over time. (D) Ex vivo 2D imaging of major organs (liver, spleen, heart, lungs, and kidneys) was performed at various time points to assess the biodistribution of the ReApoBD-BotrAMP14 nanoformulation (5 mice). 2D fluorescence intensity pattern of nanoformulation between 0 and 12 h of injection for (E) mice and (F) various organs.

In the second treatment, RAW cells infected with *S. aureus* were incubated with the ReApoBD-BotrAMP14 nanoformulation. The FITC channel revealed green fluorescent bacterial particles distributed within the intracellular space, while the 5-TAMRA signal, representing the ReApoBD-BotrAMP14 treatment, was predominantly observed at the cell periphery. This distribution suggests initial interactions between vesicles and the cell membrane, indicating early stages of internalization. The merged image demonstrated clear colocalization between bacterial particles (green) and the 5-TAMRA signal (orange), confirming the proximity of the peptide to bacteria, either at the cell membrane or within early intracellular compartments. Volumetric rendering (Figure 5B) provided a three-dimensional perspective that confirmed the spatial proximity of the peptide to bacterial particles without overlapping, suggesting localization within distinct cellular compartments.

In the third treatment (60 min treatment), the 5-TAMRA signal transitioned from the cell periphery to colocalize with bacterial particles, indicating successful intracellular delivery of the peptide (Figure 5A). Partial overlap of 5-TAMRA and FITC signals, as revealed by volumetric analysis, indicated localized peptide interaction with bacteria without cytoplasmic distribution. In the merged image, the intense green fluorescence from FITC partially masked the 5-TAMRA signal; however, faint yellow regions indicated localized interactions between the peptide and bacterial particles (Figures 5A, S9B).

### *In Vivo* Biodistribution of ReApoBD-BotrAMP14 Nanoformulation

The *in vivo* biodistribution of the ReApoBDs-BotrAMP14-NH2-Cy7 nanoformulation was evaluated using a subcutaneous injection model in BALB/c mice. Fluorescence monitoring at the injection site, in significant organs, and over time provided insights into the ReApoBDs-BotrAMP14’s systemic distribution and tissue accumulation (Figure 5C–F).

*In vivo* 2D fluorescence imaging and intensity pattern of nanoformulation between 0 and 12 h postinjection in BALB/c mice demonstrate the temporal dynamics of fluorescence at the injection site (Figure 5C,E). Initially, a high-intensity fluorescence signal was observed at the injection site immediately after administration (0 h), confirming the successful delivery of the ReApoBDs-BotrAMP14 nanoformulation. This signal steadily decreased over time, and by 12 h, fluorescence at the injection site was undetectable, indicating complete dispersion or clearance from the delivery site. Figure 5D,F provide complementary insights into the accumulation of ReApoBD-BotrAMP14 treatment in major organs. Quantitative analysis reveals a redistribution of fluorescence from the injection site to the systemic circulation, peaking in key metabolic organs, such as the liver and kidneys, by 3–6 h (Figure 5F). These organs play central roles in metabolism and excretion, confirming their involvement in the clearance and processing of the ReApoBDs-BotrAMP14 nanoformulation. Fluorescence in the lungs peaked at 6 h, likely due to transient circulation through the pulmonary vasculature. Minimal fluorescence was detected in the spleen and heart, suggesting limited interaction with these organs.

For instance, in rabbit models of endocarditis and osteomyelitis, SCVs persisted in the kidneys and spleen despite oxacillin treatment, highlighting the difficulty of eradicating these bacterial subpopulations in systemic infections.^62^ Notably, our biodistribution analysis revealed renal accumulation of the ReApoBD-BotrAMP14 nanoformulation, an essential advantage given that SCVs are known to persist in the kidneys. This targeted accumulation, combined with the demonstrated potency of ReApoBD-BotrAMP14 against SCVs, reinforces its potential as a therapeutic strategy for persistent *S. aureus* infections. Furthermore, the nanoformulation’s dynamic redistribution and gradual clearance suggest a favorable pharmacokinetic, particularly when compared to conventional antibiotic treatments that often fail to effectively clear SCVs from organs such as the kidneys and spleen.^59,62^ Importantly, the concentrations used in both the biodistribution and efficacy experiments had been previously confirmed to be locally well tolerated in a preliminary *in vivo* tolerability assessment, with no visible signs of tissue damage at the injection site. Moreover, under the tested conditions—a single low dose (0.3 mg kg^–1^) and short-term monitoring—no evident clinical signs of systemic toxicity or distress (e.g., weight loss, behavioral changes, or visible organ impairment) were observed, indirectly supporting a favorable safety profile of the nanoformulation. This improved biodistribution profile supports its potential for systemic application in persistent *S. aureus* infections.

## Conclusions

This study introduces a novel nanoformulation using ReApoBDs conjugated with the antimicrobial peptide BotrAMP14, representing a potentially valuable strategy for tackling persistent intracellular infections. Designing antimicrobial treatments for intracellular infections requires a careful balance between the efficacy of the drug and its cytotoxicity to host cells. While direct intracellular delivery of antimicrobial agents enhances therapeutic outcomes, it also carries the risk of off-target effects or disruption of essential cellular functions. Our findings highlight the importance of assessing cytotoxicity when developing intracellular antimicrobial treatments. Compared to conventional free-drug administration, nanoformulations such as ReApoBDs offer a promising alternative by modulating drug release and reducing toxicity. Future studies should explore the long-term effects of ReApoBD-based formulations on host cell function and their potential for targeted intracellular delivery mechanisms that optimize therapeutic efficacy while minimizing adverse effects.

The *in vitro* antimicrobial efficacy of ReApoBDs was significant, with ReApoBDs-VAN and ReApoBDs-BotrAMP14 achieving substantial intracellular bacterial clearance. The proposed mechanism of action (Figure 6) provides a possible explanation for these effects: (i) fusion of the endosome containing the nanoformulation with the phagolysosome, allowing direct interaction with intracellular bacteria, and (ii) direct cytosolic action of the nanoformulation following bacterial escape from the phagolysosome. The first hypothesis suggests an efficient antimicrobial response by targeting *S. aureus* within its intracellular niche. In contrast, the second mechanism could explain the observed cytotoxic effects, as the formulation’s presence in the cytosol might inadvertently impact host cell viability. Given that SCVs persist intracellularly by adapting to the phagolysosomal environment, these mechanisms of action could enhance their eradication by directly disrupting these adaptations (Figure 6).

**Figure 6:**
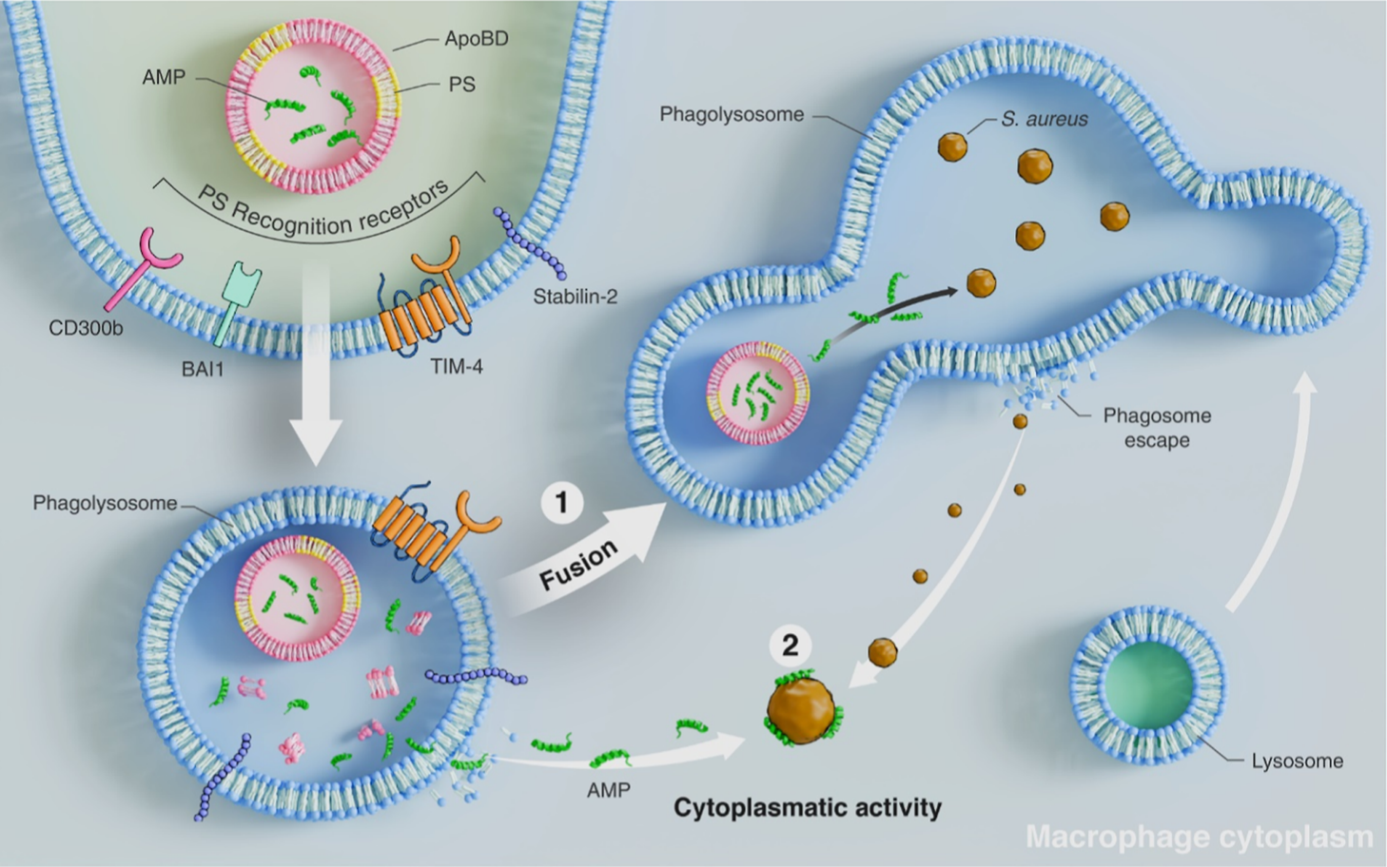
Scenario of a macrophage with intracellular infection caused by *S. aureus* and treatment mechanism of action with a cell-derived nanocarrier formulation based on apoptotic bodies-antimicrobial peptide (e.g., ApoBDs-BotrAMP14). The mechanism of internalization of ApoBDs is illustrated, where they release their content inside phagosomes, followed by two hypotheses regarding the mechanism of action of ApoBDs-AMP formulations. The first hypothesis is based on the fusion between the endosome containing the formulation and the phagolysosome. The second is based on the direct action of the formulation in the cytosol once the bacteria have escaped from the phagolysosome. Both hypotheses could co-occur.

While our study provides valuable insights into the potential of ApoBD-based cell-derived nanocarriers formulations for delivering antimicrobial agents, several limitations must be acknowledged. The variability in encapsulation efficiency and drug release profiles, influenced by donor cell type and vesicle characteristics, underscores the need for further optimization. Differences in membrane composition, such as cholesterol content and surface charge, affected drug entrapment and stability, with HeLa-derived ApoBDs favoring vancomycin encapsulation due to their higher cholesterol levels, while BV-2-derived ApoBDs were more efficient for BotrAMP14 encapsulation due to their lower negative charge. These factors must be considered to ensure consistent therapeutic outcomes. The lack of direct evidence for drug-release mechanisms within phagolysosomes also limits our understanding of the intracellular targeting process. Future studies should further validate the findings in expanded animal models of *S. aureus* infections to address these challenges, with a focus on pharmacokinetics, biodistribution, and long-term efficacy. Moreover, the scalability and reproducibility of ApoBD production need to be established, as these factors are critical for clinical translation. While the concept holds translational promise, further experimental work is required to assess its real-world applicability. These steps will be essential for bridging the gap between laboratory findings and real-world clinical applications, ultimately advancing treatment strategies for persistent bacterial infections.

Taken together, our results support the use of ReApoBD-BotrAMP14 as a promising intracellular antimicrobial strategy, particularly against persistent *S. aureus* infections. The observed reduction in abscess size, total bacterial burden, and SCV frequency highlights its multifaceted antimicrobial potential. These findings lay a foundation for future research into the use of cell-derived vesicles for targeting complex intracellular bacterial populations.

## Material and Methods

### Cell Culture and Bacterial Strain

BV-2, HeLa, and RAW 264.7 cell lines were purchased and cultured according to the protocols of the Rio de Janeiro Cell Bank (BCRJ) and the Instituto Adolfo Lutz (IAF). BV-2 cells, derived from mouse microglial cells immortalized with v-raf/v-myc oncogenes, and HeLa cells from human cervical cancer, were maintained in DMEM or RPMI medium supplemented with 10% fetal bovine serum (FBS) at 37 °C in a 5% CO_2_ atmosphere. *S. aureus* strain Aurora 457 (Universidade Federal de Mato Grosso do Sul, Campo Grande, Mato Grosso do Sul, Brazil), a clinical isolate from dairy cows with mastitis symptoms, was used to infect RAW 264.7 mouse macrophage cells to assess the antibiotic effects of treatments (Figure S10).

### Induction of Apoptosis

BV-2 and HeLa cell lines were thawed, grown in culture flasks to approximately 80% confluence, and then detached. Next, ∼2 × 10^6^ viable cells per well were plated into a 6-well culture plate and incubated at 37 °C for 24 h to allow adherence. Subsequently, the culture medium was removed, and each well was washed three times with 1× phosphate-buffered saline solution (PBS) (pH 7.4). Finally, RPMI culture medium without FBS^63^ and 0.09% hydrogen peroxide (H_2_O_2_)^27^ were added to each treatment well to induce the apoptosis process. Using an inverted Zeiss microscope, the cell culture was monitored every 12 h, recording the presence of apoptotic morphologies such as blebbing, apoptopodia, and ApoBD formation at 40× magnification. Additionally, Hoechst 33342 dye (Thermo Fisher Scientific, USA) was used to stain nuclei, enabling the visualization and confirmation of apoptotic cells by fluorescence microscopy.

### Isolation of ApoBDs

Following the confirmation of apoptosis induction, ApoBDs were isolated from other cellular particles and debris present in the culture suspension, commonly achieved via differential centrifugation. We followed a previously described protocol for detecting and separating high-purity ApoBDs with some modifications.^64^ Briefly, the contents of all apoptosis-inducing wells were collected, and three tubes were prepared. The first tube contained the control sample of viable cells, the second one contained all apoptosis-induced cells, and the third one contained 1/10th of the whole apoptotic sample (WAS). The first and third tubes were centrifuged at 3000*g* for 6 min. The pellets were resuspended in 2 mL of 1× PBS and kept on ice. The second tube was centrifuged at 300*g* for 10 min to separate apoptotic cells from other extracellular vesicles (EVs) and debris. The supernatant was transferred to a new tube labeled “ApoBDs”, and the remaining pellet-containing tube was labeled “apocells”. The pellet was resuspended in 3 mL of 1× PBS and stored on ice alongside the WAS and viable cells as controls. Finally, the ApoBD tube was centrifuged at 3000*g* for 20 min, and the supernatant was discarded. The formation of the pellet was visually confirmed, and it was resuspended in 3 mL of 1× PBS. The three control and the ApoBD tubes were used to verify the separation of different populations via flow cytometry.

#### Confirmation of Apoptotic Bodies Isolation by Flow Cytometry

FC was used to confirm the separation of ApoBDs after the initial application of the differential centrifugation method^64^ with some modifications. Briefly, 100 μL of the previously separated samples were taken and placed into four new round-bottomed polystyrene FC tubes. Then, 100 μL of a staining solution containing 2 × A5 binding buffer and 1:100 A5-FITC was added to each tube. The tubes were then incubated for 10 min at room temperature in the dark. The samples were measured on a FacsCanto II flow cytometer (BD Biosciences, San Jose, CA, USA) using FACS Diva V6.0 software (BD Biosciences). The cytometer machine was previously calibrated using BD Cytometer Setup and Tracking beads (BD Bioscience) to adjust the fluorescence and light scattering detectors. Once calibrated, the equipment was configured with a 100 μm nozzle and drop delay, ensuring a stable flow rate and setting the acquisition speed to ∼1000 events.s^–1^. After adjusting the FSC, SSC, and FITC (A5) voltages, events were positioned within each plot, and 20.000 events were recorded per run.

##### Field Emission Transmission Electron Microscopy

For sample TEM analysis, carbon-coated 300 square copper mesh grids were glow-discharged using an EMS GloQube, dual chamber glow-discharge system (Electron Microscopy Sciences, Hatfield, PA, USA) in negative mode at a plasma current of 15 mA for 120 s. A 10 μL drop of the apoptotic cell or ReApoBD suspensions was applied to the grids for 1 min, and the excess sample was removed with absorbent paper. A 10 μL drop of 1% phosphotungstic acid (PTA) was added to the grids, and the excess was blotted off. The grids were examined using a JEOL JEM-1400 Flash transmission electron microscope (JEOL, Tokyo, Japan) at 120 kV acceleration voltage.

#### Peptide Synthesis and Validation

The BotrAMP14 was synthesized using F-moc solid-phase chemical synthesis by AminoTech Research & Development Ltd.a., São Paulo, Brazil. Its purity, confirmed to be over 95%, was verified through reverse-phase high-performance liquid chromatography (RP-HPLC) (LC system with an LC-20AR pump, SIL-10AF autoinjector, and SPD-M40 photodiode array detector Shimadzu Corp., Japan) and mass spectrometry (MALDI-ToF Ultraflex III, Bruker Daltonics, USA).

#### Loading of Therapeutic Agents

Both vancomycin hydrochloride (VAN) (from Streptomyces orientalis, Sigma-Aldrich, USA) and BotrAMP14 at a 1:1 v/v ratio (250 μg mL^–1^) were mixed with isolated ReApoBDs (78 μg mL^–1^ protein equivalent quantified by Bradford, (1976).^65^ Moreover, they were subjected to three freeze–thaw cycles of −20 °C/20 min and 45 °C/10 min. A sequential extrusion process was performed using an Avanti Mini Extruder to ensure uniform vesicle sizing through 0.8, 0.4, 0.2, and 0.1 μm pore polycarbonate membranes.^12^ This resulted in the final nanoformulated ReApoBDs (∼100 μm ApoBDs) with VAN and BotrAMP14 or ReApoBDs-VAN and ReApoBDs-BotrAMP14, respectively.

#### Encapsulation Efficiency of Therapeutic Agents

The encapsulation efficiency (EE) of VAN and BotrAMP14 on the ApoBDs was quantified using RP-HPLC. Chromatographic separation was achieved on a Venusil ASB C18 column (250 mm × 4.6 mm × 5 μm particle size, Bonna-Agela Technologies) using a mobile phase gradient of water and acetonitrile. The flow rate was set at 1.0 mL.min^–1^, and VAN and BotrAMP14 were detected at 230 and 216 nm, respectively. Calibration curves were generated for both compounds (each time a quantification assay was performed) using known concentrations of each to allow quantification of the amounts encapsulated in the ApoBDs. Only equations with *R* ^2^ greater than or equal to 0.9 were used for quantification each time. The %EE was calculated using eq 1, where the “free compound” is the amount of unencapsulated drug or peptide determined in the supernatant postultrafiltration (Amicon Ultra-4 centrifugal filters, ten kDa). After filtration, the ReApoBD-VAN and ReApoBD-BotrAMP14 fractions were mixed with a solution containing 0.1% Triton X-100 in PBS to lyse the vesicles, and the amount of peptide released was quantified to determine the total amount of formulated peptide. The amount of encapsulated peptide was determined, and both methods yielded values with differences of less than 5%.

1 $$\%EE=\left(\frac{free\;compound}{Total\;compound\;added}\right)\times 100\%$$

The mass of the recovered VAN and BotrAMP14 was confirmed by mass spectrometry using matrix-assisted laser desorption ionization time-of-flight (MALDI-ToF).

#### Drug Release of Therapeutic Agents

The drug release was evaluated using the RP-HPLC quantification methods described above. Formulations were prepared at the highest quantifiable concentration using the calibration curve equation for each drug and stored at room temperature. Aliquots were taken at 0, 2, 4, 8, 12, and 48 h, centrifuged (Amicon Ultra-4 centrifugal filters, 10 kDa, prerinsed with buffer to minimize adsorption), and the volume was adjusted not to alter the concentration measurement. RP-HPLC was used to determine the amount of drug released at each time point analyzed. Additionally, release was assessed in a lysosomal-mimicking buffer (pH 5.0 with 10 mM GSH).^41^ For this condition, samples were incubated at 37 °C under orbital agitation (100–150 rpm) in sink conditions (release medium/formulation 10:1, with medium replacement when necessary). Aliquots were collected at the same time points, including an early sampling at 30 min to capture potential rapid release. Ultrafiltration was employed to separate the initially free peptide fraction from the vesicle-associated fraction, and peptide-only controls in both PBS and pH 5 + 10 mM GSH were included to monitor release and stability during analysis.

#### Dynamic Light Scattering and Zeta Potential Measurement

The size distribution and electrokinetic potential of the formulated ReApoBDs were analyzed using a Zetasizer Pro (Malvern Instruments, UK). The pure empty ReApoBDs and formulated suspensions were centrifuged, washed 2 times, and diluted in 0.1× PBS. DTS0012 and DTS1070 cells were used for DLS and ζ potential measurements. Measurements were performed in triplicate per sample with a minimum of 15 runs each, at 25 °C, pH 7.4, and a voltage level of 150 V for the ζ potential. The ζ potential was measured at different time points (0, 2, 4, 8, 12, and 48 h) to evaluate the relative colloidal stability of the drug formulations by comparing the value at each time point (*t*_*x*_) with that at time 0 (*t*_0_) according to eq 2. Additionally, to assess freeze–thaw stability, ReApoBDs-VAN and ReApoBDs-BotrAMP14 formulations were stored in PBS at – 80 °C and analyzed for 5 days using the same DLS and ζ-potential protocols described above. For the lysosomal-mimicking condition, samples were incubated in pH 5.0 buffer supplemented with 10 mM GSH, and ζ potential was measured at the same time intervals (including 30 min) to monitor the impact of acidic and reductive environments on colloidal stability. The data were processed using the absorption and refractive indices of the liposomes, 0.001 and 1.45, respectively, and analyzed using ZS Xplorer software v. 2.2.0.147, which provides information on the mean particle size (hydrodynamic diameter), polydispersity index (PDI), and ζ potential of the formulates.

2 $$Relative\;stability=1-\left(\frac{\left|\overset{®}{t_{x}\;}-\overset{®}{t_{0}\;}\right|}{\overset{®}{t_{0}\;}}\right)$$

#### Atomic Force Microscopy

An SPM-9700 Atomic force microscope in dynamic mode (Shimadzu Corp., Japan) was used for topographic imaging of the vesicles. Clean cover glass slides were attached to AFM/scanning tunneling microscope (STM) magnetic stainless-steel specimen discs using double-sided tape. At room temperature, the top surface of the slides was treated with a 0.5% gelatin solution. After 24 h, the ApoBD and ReApoBD samples were diluted 200-fold in ultrapure water, and a 50 μL drop of the solution was applied to the gelatin surface. The STM discs with the samples were incubated for ∼18 h at 18 °C in a glass desiccator. Before imaging, the sample surfaces were rinsed 3 times with ultrapure water and allowed to dry. The noncontact cantilevers (Nanoworld, Switzerland) used for imaging had dimensions of 4 μm thickness, 125 μm length, and 30 μm width, and a resonant frequency of 320 kHz and a force constant of 42 N.m^–1^ were used. Trace and retrace scans and topographic heights were imaged at a 0.5 to 1 Hz scanning rate, with images recorded at 512 × 512 pixels and processed using SPM analysis software (Shimadzu Corp., Japan).

#### Determination of Minimum Inhibitory Concentration

The VAN and BotrAMP14 MIC were assessed using a broth microdilution method by the Clinical & Laboratory Standards Institute (CLSI) guidelines (protocol M07-A10). To the mid log phase, *S. aureus* Aurora was cultured in Mueller–Hinton broth (MHB). Serial 2-fold dilutions of VAN (32 to 0.25 μM) and BotrAMP14 (256 to 2 μM) were prepared in 96-well plates. Bacteria were added to each well, and the plates were incubated at 37 °C for 18 h. Finally, the lowest concentration that inhibited bacterial growth was reported as the MIC and determined by reading the plates at OD_600_.

#### Cytotoxicity Essays

The MTT assay determined the lethal and sublethal concentrations of VAN, BotrAMP14, empty ReApoBDs, and nanoformulations against RAW 264.7. Macrophages were grown in 96-well plates at a density of 3.5 × 10^3^ cells per well. Serial 2-fold dilutions of VAN/ReApoBDs-VAN (32 to 0.25 μM), BotrAMP14/ReApoBDs-BotrAMP14 (256 to 2 μM), and empty ReApoBDs (78 μg mL^–1^) were added to the wells and incubated at 37 °C for 24 h. Then 10 μL of MTT (5 mg.mL^–1^) was added to each well, and the plate was incubated at 37 °C for 6 h with constant shaking and protected from light. A hundred μL of isopropanol was added to each well to solubilize the formed formazan crystal. Finally, the OD_570_ nm was read, and the cell viability was calculated according to eq 3

3 $$Cell\;viability\;\%=\frac{optical\;density\;of\;treated\;cells}{optical\;density\;of\;untreated\;cells}\times 100\%$$

#### *In Vitro* Antibacterial Efficacy Testing

RAW 264.7 macrophages were cultured and infected with *S. aureus* at a multiplicity of infection (MOI) of 10 (i.e., ten bacterial cells per macrophage). The culture plates were centrifuged at 600*g* for 5 min to allow bacterial cells to settle, followed by a 2 h incubation period for phagocytosis. Macrophage cells were washed 10 times with 1× PBS and treated with 200 μg mL^–1^ gentamicin (Sigma-Aldrich, USA) to eliminate extracellular bacteria for 6 h.^12,66^ The culture medium was then changed back to an antibiotic-free medium. Control groups were established, using uninfected cells to determine baseline cell health and for statistical comparison. Infected, untreated cells served as a comparison for treatment efficacy. The efficacy of free and formulated VAN and BotrAMP14 was evaluated over a concentration range of 256–0.25 μM and 256 to 2 μM, respectively. After 24 h of treatment, 100 μL of medium aliquots were carefully collected to avoid detaching the macrophages and plated on Mueller–Hinton agar (MHA) to determine the extracellular bacterial concentration. The remaining samples with macrophages were then lysed with a solution containing 0.1% Triton X-100 in PBS to release intracellular bacteria. To remove cellular debris, the lysates were centrifuged at 300*g* for 5 min. The bacteria were then separated by centrifugation at 5000*g* for 5 min and resuspended in 1× PBS.^67^ Serial dilutions of the bacterial samples were then plated on MHA to determine the total *S. aureus* concentration. Finally, the extracellular and reduced intracellular bacterial load (total bacteria – extracellular bacteria) attributable to each treatment was compared to the infected, untreated control.

#### Intracellular Uptake Assay Using Confocal Microscopy

RAW 264.7 cells were seeded on 6-well plates and grown on sterilized glass slides for 24 h. *S. aureus* bioparticles (*S. aureus* Wood strain without protein A BioParticles, fluorescein-FITC conjugate, Thermo Fisher Scientific, USA, catalog #S-2851) at a MOI of 100 were added to the 6-well plate. The plate was centrifuged at 600*g* for 5 min at room temperature to allow the particles to settle, followed by a 2 h incubation period to facilitate phagocytosis. After bioparticle incubation, the cells were washed 3 times, and ReApoBDs-BotrAMP14-NH2-TAMRA (78 μg mL^–1^–64 μM) was added to the wells to evaluate the phagocytosis of the vesicles for peptide internalization, and incubated for 20, and 60 min. The cells were washed 5 times with prewarmed PBS, fixed with 4% formaldehyde, washed 3 times, and permeabilized with 0.1% Triton X-100. Finally, DAPI staining was performed with a 0.25 μg mL^–1^ solution for ∼3 min, followed by 3 PBS washes. Slides were analyzed using a Zeiss LSM 900 microscope with a 63× objective, and the images were analyzed using the ZEISS arivis Pro software (version 4.1.2) to evaluate peptide localization.

#### *In vivo* Bacterial Killing Assay

To assess the antimicrobial efficacy of the ReApoBD-BotrAMP14 nanoformulation, an *in vivo* cytotoxicity assay was first performed to determine the maximum concentration that could be used without causing tissue toxicity. For this, increasing concentrations of BotrAMP14 and ReApoBD-BotrAMP14 (8 to 64 μM), as well as 78 μg mL^–1^ of vesicles (ReApoBDs), were injected subcutaneously into the back of mice. After 24 h, the tissue condition was evaluated to confirm that the selected concentrations were well tolerated before proceeding with the antimicrobial efficacy study.

The *in vivo* antimicrobial efficacy of the peptide formulation was evaluated using a mouse abscess model.^68^ All animal studies were conducted using a protocol approved by the University of Otago Animal Ethics Committee under approval number AUP-19–125. Swiss Webster (SW) male and female mice (6–8 weeks old) purchased from the University of Otago Biomedical Research Facility were used to evaluate the antibacterial effect of the ReApoBDs-BotrAMP14 nanoformulation. A skin infection model was used with 14 mice per group, divided into three biological replicates conducted at different times. The mice were shaved before being injected subcutaneously in their back with 1 × 10^8^ CFU *S. aureus* Aurora to induce abscess formation. After 4 h of infection, the animals were treated with either saline or the formulation (78 μg mL^–1^–64 μM, 50 μL). Mice were euthanized by an inhalant anesthetic overdose followed by cervical dislocation after 3 days. The abscess area was measured, the surrounding non-necrotic skin was collected, homogenized, and a solution containing 0.1% Triton X-100 in PBS was added to release intracellular bacteria. Serial dilutions of the bacterial samples were plated on MHA to determine the total *S. aureus* concentration.

Small colony variants (SCVs) were identified based on their distinctive morphology on MHA plates (small, nonpigmented, and nonhemolytic colonies), in accordance with previously described diagnostic criteria.^57^ Additionally, suspected SCVs were subcultured in fresh media to assess their ability to revert to the parental (larger) phenotype, further supporting their identification.^9^

#### *In Vivo* Biodistribution

All animal studies were conducted in accordance with a protocol approved by the Universidade Católica Dom Bosco Animal Ethics Committee, under approval number 017/2021. BALB/c female mice (6–8 weeks old), purchased from the Universidade Estadual de Campinas (UNICAMP, Brazil), were used to evaluate the biodistribution of the ReApoBDs-BotrAMP14-NH2-Cy7 nanoformulation.^69,70^ Five mice were used: one untreated control injected with saline solution and four treated with the ReApoBD-BotrAMP14-CY7 nanoformulation, each corresponding to a specific time point (1, 3, 6, and 12 h) for organ extraction. The mice were shaved and injected subcutaneously with the nanoformulation (78 μg·mL^–1^, 64 μM, 50 μL) into the back before imaging. After 1, 3, 6, and 12 h of treatment, organs (i.e., liver, spleen, kidneys, heart, and lungs) were collected from the treated mice and compared with the corresponding organs from the untreated control animal. Fluorescence imaging was performed using the Newton FT500 with an X54276 camera and a 24 mm motorized lens. Calibration steps included evaluating signal linearity using known ReApoBD-BotrAMP14-CY7 concentrations (256 to 8 μM), focusing on reproducibility and sensitivity. The system was optimized for Cy7 detection, with the excitation source set to 780 nm and the emission set to 800 nm, thereby eliminating autofluorescence. Fluorescence intensity was expressed in physical units of radiance (photons per second per square centimeter of the sample and per steradian of solid angle detected by the system). These units enable a quantitative comparison of fluorescence signals between samples while accounting for variations in illumination and detection geometry. The exposure and aperture were adjusted to ensure accurate detection without oversaturation, and these parameters were maintained at constant levels throughout the experiment to facilitate direct comparisons between samples. The images were processed using Kuant software (version 2.3) to quantify fluorescence signals. Quantification was performed by integrating the fluorescence intensity over a region of interest (ROI) corresponding to each organ, normalized to the control fluorescence background to account for potential autofluorescence.

#### Statistical Analysis

The statistical analyses were performed in R (R core team, Version 4.4.2). Data distribution was determined by the Shapiro–Wilk test. Students’ *t* tests evaluated the differences between treatments and controls. The reduction in the *in vitro* intracellular bacterial load was analyzed using ANCOVA, with macrophage viability as a covariate to account for the potential cytotoxic effects of the treatments. Statistical significance was set a priori at *p* < 0.05.

## Supplementary Material



## Notes

Supplementary figures and tables related to the experimental design, apoptosis induction, drug encapsulation and release, nanoparticle characterization, cytotoxicity evaluation, and intracellular uptake analysis are available. This includes: Figures S1–S10: apoptosis induction, peptide/drug quantification, encapsulation efficiency, drug release and stability, freeze–thaw stability, correlation and phase plot, vesicle size distribution, cytotoxicity mechanisms, confocal imaging of intracellular uptake, and experimental workflow. Table S1–S2: dynamic light scattering (DLS), zeta potential, and encapsulation efficiency of ReApoBD nanoformulations and minimum inhibitory concentrations (MIC) values for free compounds and nanoformulations (PDF)

## Abbreviations

AFM: atomic force microscopy
AMP: antimicrobial peptide
ApoBDs: apoptotic bodies
CB-tag: coomassie blue–cholesterol tag
DAPI: 4′,6-diamidino-2-phenylindole
EE: encapsulation efficiency
FITC: fluorescein isothiocyanate
FSC: forward scatter
HPLC: high-performance liquid chromatography
ICAM-3: intercellular adhesion molecule 3
MALDI-ToF: matrix-assisted laser desorption/ionization time-of-flight
MIC: minimum inhibitory concentration
MOI: multiplicity of infection
MTT: 3-(4,5-dimethylthiazol-2-yl)-2,5-diphenyltetrazolium bromide
PBS: phosphate-buffered saline; PC, phosphatidylcholine
PDI: polydispersity index
PS: phosphatidylserine
ReApoBDs: size-remodeled apoptotic bodies
RP-HPLC: reverse-phase high-performance liquid chromatography
SCVs: small colony variants
SSC: side scatter
TAMRA: carboxytetramethylrhodamine
TEM: transmission electron microscopy
VAN: vancomycin
ζ-potential: zeta potential.
