# Supplementary material for: Cell-Derived Nanocarriers of Apoptotic Bodies with an Antimicrobial Peptide for Targeting Intracellular S. aureus Infections
Source: ACS Appl Bio Mater. 2025 Oct 22;8(11):9875–92. doi: 10.1021/acsabm.5c01222 (PMC12628330; doi:10.1021/acsabm.5c01222)

Supporting Information

Cell-Derived Nanocarriers of Apoptotic Bodies with an Antimicrobial Peptide for Targeting Intracellular *S. aureus* Infections

Valentina Nieto-Marín[1,5], Ian Alejandro Fernandez-Soliz[1], Jorge William Arboleda Valencia[2,3,4], Daniel Pletzer[5], Danieli Fernanda Buccini[1], Octávio Luiz Franco[1,6,*].

[1] S-Inova Biotech, Programa de Pós-Graduação em Biotecnologia, Universidade Católica Dom Bosco, Campo Grande, MS, Brazil.
[2] Grupo de Investigación FITOBIOL, Instituto de Biología, Facultad de Ciencias Exactas y Naturales, Universidad de Antioquia, Medellín, Antioquia, Colombia
[3] Laboratorio HERSSEN - Grupo de Investigación en Medicina Integrativa y Fitomedicina, Manizales, Caldas, Colombia
[4] Centro de Investigaciones en Medio Ambiente y Desarrollo-CIMAD, Universidad de Manizales, Manizales, Caldas, Colombia
[5] Department of Microbiology and Immunology, School of Biomedical Sciences, University of Otago, Dunedin, Otago, New Zealand.
[6] Centro de Análises Proteômicas e Bioquímicas, Programa de Pós-Graduação em Ciências Genômicas e Biotecnologia, Universidade Católica de Brasília, Brasília, DF, Brazil.

This PDF file includes:

Figs. S1 to S10
Tables S1 and S2

Fig. S1.

Induction of apoptosis in BV-2 cell cultures (**A**). Before and (**B**). 72 h after induction of apoptosis by starvation and H2O2 treatment. Images show the presence of ApoBDs (floating particles, black arrow), apoptotic cells (red arrow), and other cell debris and EVs.

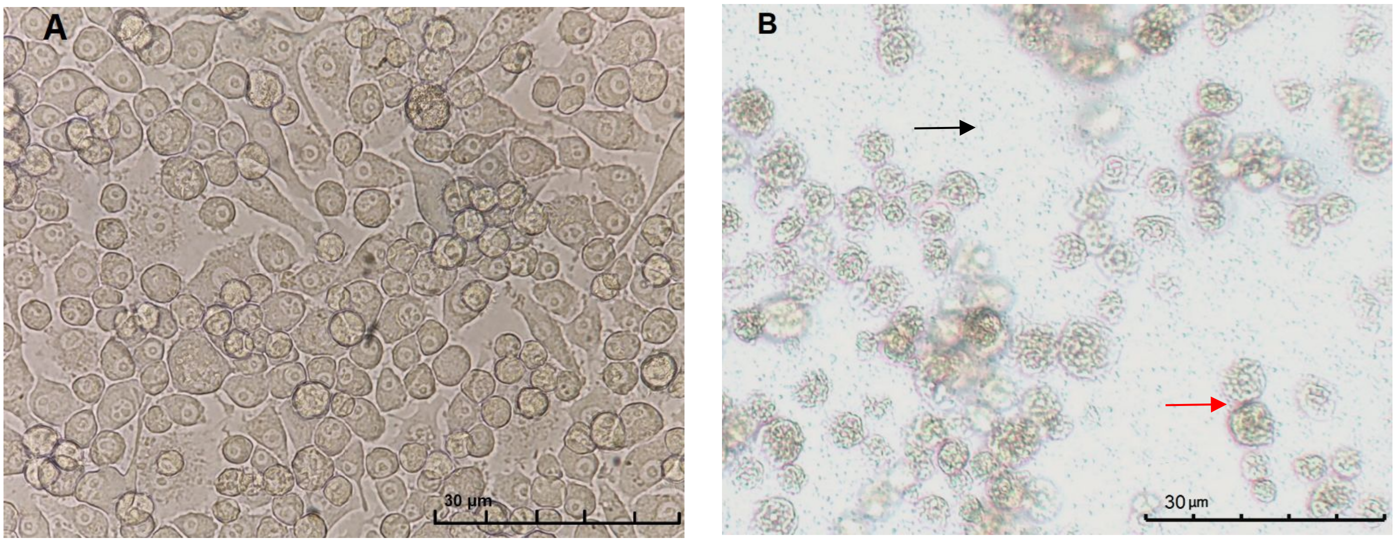

Fig. S2.

Detection and quantification of vancomycin and BotrAMP14 by RP-HPLC and MALDI-ToF. Chromatograms of drug detection (64 µM), calibration curves, and equations examples for %EE quantification of (**A, B**) vancomycin and (**C, D**) BotrAMP14, respectively. The %EE was determined by subtracting the amount of free VANH or BotrAMP14 from the total amount initially mixed with the ApoBDs and verified by quantification of the encapsulated peptide after lysis of the nanoconjugate vesicles. (**E**). Mass spectrum of vancomycin and BotrAMP14 stocks and post-RP-HPLC recovery fractions from one of the experiments, showing an ion mass of approximately 1471 Da and (**F**) 1957.51 Da, respectively.

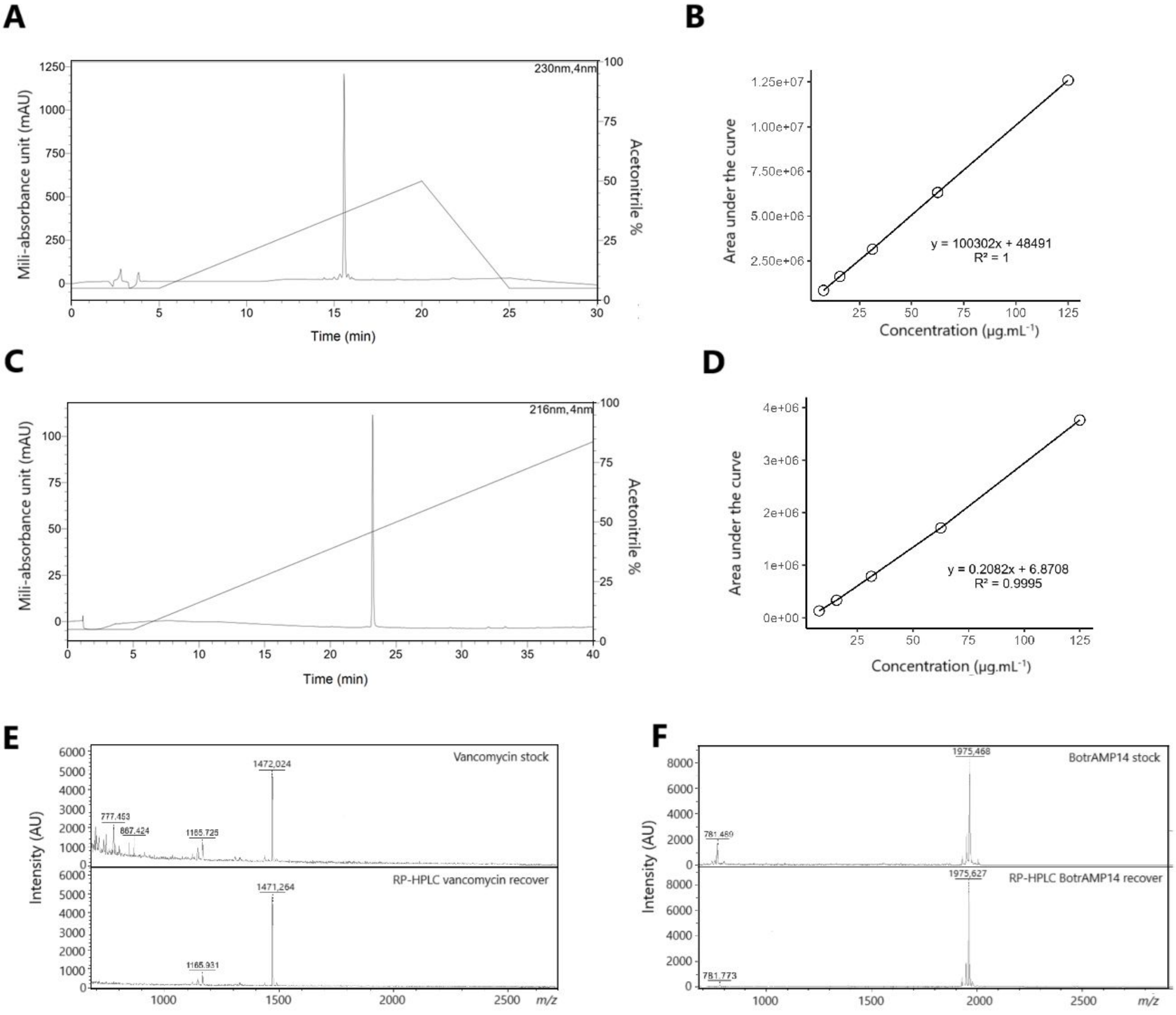

Table S1.

Dynamic light scattering (DLS) and encapsulation efficiencies of ApoBDs and ReApoBDs nanoformulations with VAN or BotrAMP14. The nanoformulations were prepared from HeLa and BV-2 cell cultures using freeze-thaw and freeze-thaw combined with extrusion as encapsulation methods. * Indicates cell populations that are highly heterogeneous or outside the measurable size and zeta potential range, where only the data from the two most similar technical replicates were used. Data represent the mean ± SD of at least three replicates.

Particle/Formulation	Hydrodynamic diameter (nm)	Surface charge (mV)	Polydispersity index (PDI)	Encapsulation efficiency (%EE) w/wo extrusion
HeLa Apocells *	5049 ± 4165	ND	ND	
Empty HeLa ApoBDs *	2911 ± 1732	-27.1 ± 5.83	0.452	
Empty HeLa ReApoBDs	97.5 ± 4.7	-35 ± 3.84	0.14	
HeLa ReApoBDs+VAN	117.9 ± 11.16	-28 ± 6.13	0.14	45.75 ± 0.63 / 17.53 ± 2.11
HeLa ReApoBDs+BotrAMP14	93.74 ± 12.35	-17.3 ± 6.22	0.457	22.25 ± 0.89 / 2.23 ± 0.67
BV-2 Apocells *	5275 ± 4054	ND	ND	
Empty BV-2 ApoBDs *	2999 ± 1962	-23.4 ± 4.17	0.484	
Empty BV-2 ReApoBDs	94.44 ± 3.1	-31.45 ± 4.28	0.181	
BV-2 ReApoBDs+VAN	119.73 ± 13.12	-25.46 ± 4.57	0.224	17.56 ± 1.41 / 0.63 ± 0.2
BV-2 ReApoBDs+BotrAMP14	86.86 ± 6.1	-21.4 ± 4.05	0.202	70.8 ± 3.39 / 5.32 ± 2.55

Fig. S3.

Percent encapsulation efficiency for HeLa and BV-2 formulations using freeze-thaw and freeze-thaw combined with extrusion as encapsulation methods. ApoBDs or size-modified ApoBDs (ReApoBD) from BV-2 or HeLa cultures were used for vancomycin (VAN) loading. Data represent mean ± SD of three replicates.

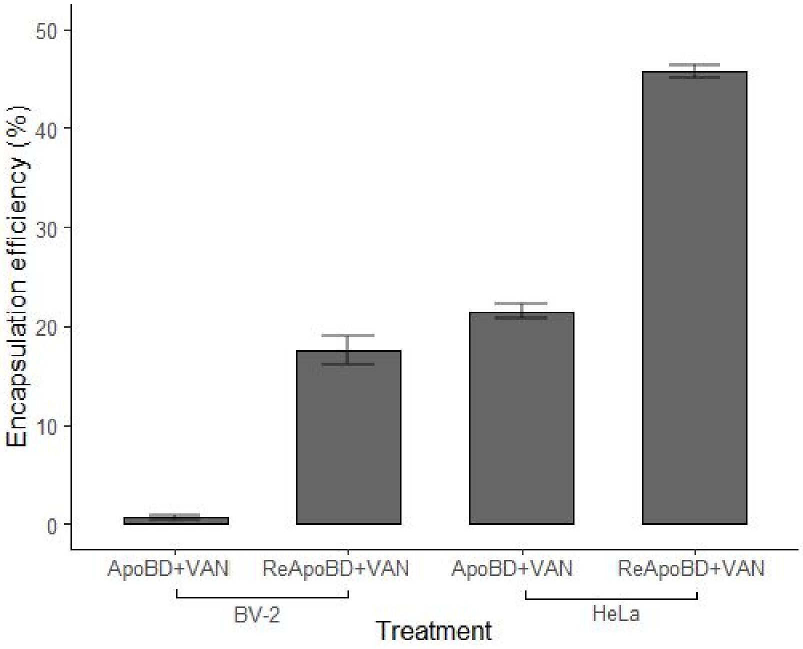

Fig. S4.

Drug release and colloidal stability of ReApoBD–BotrAMP14 in lysosomal-mimicking conditions. Release kinetics and relative stability of ReApoBD–BotrAMP14 were evaluated in a lysosomal-mimicking buffer (pH 5.0 supplemented with 10 mM glutathione). Data represent mean ± SD of three independent experiments.

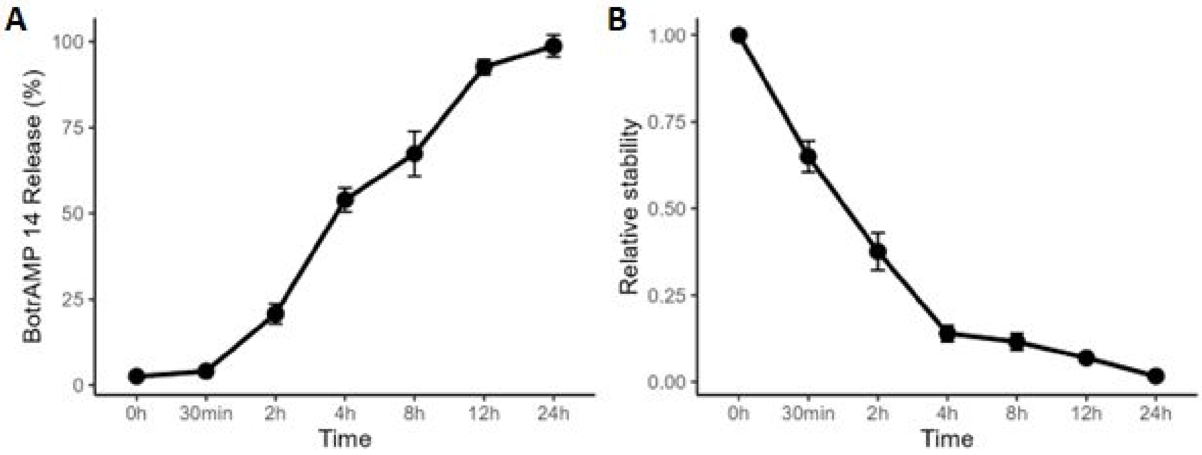

Fig. S5.

Freeze-thaw stability of ReApoBD nanoformulations. Relative stability of ReApoBD-BotrAMP14 and ReApoBD-VANH nanoformulations stored in PBS at –80 °C over 5 days. Freeze-thaw stability was calculated from ζ-potential measurements normalized to baseline values (day 0). Data represent mean ± SD of three independent experiments.

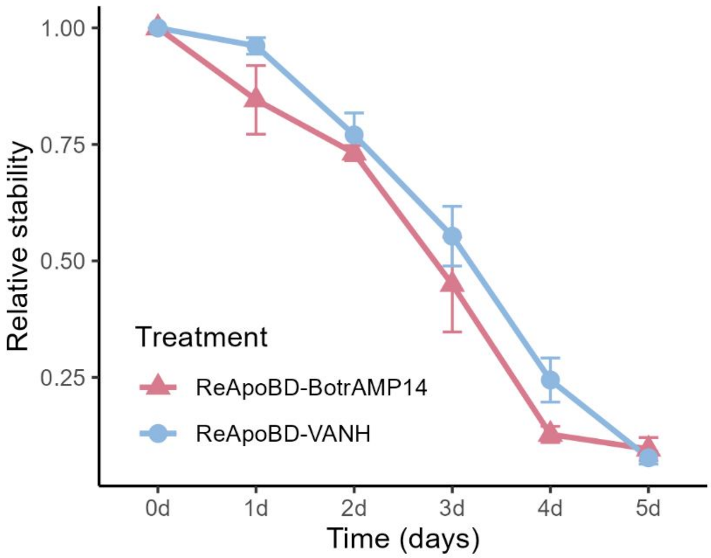

Fig. S6.

Correlation and phase plot from (**A**). size and (**B**). Zeta potential measurements.

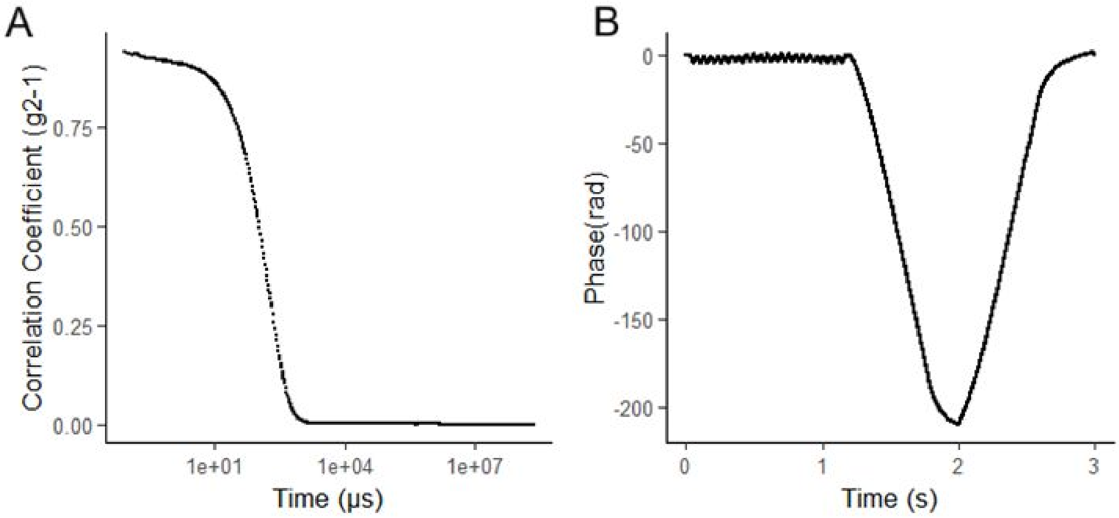

Fig. S7.

Size frequency distribution graph of different vesicles/cell populations by intensity, number, and volume. The plots represent the data distribution of three replicates.

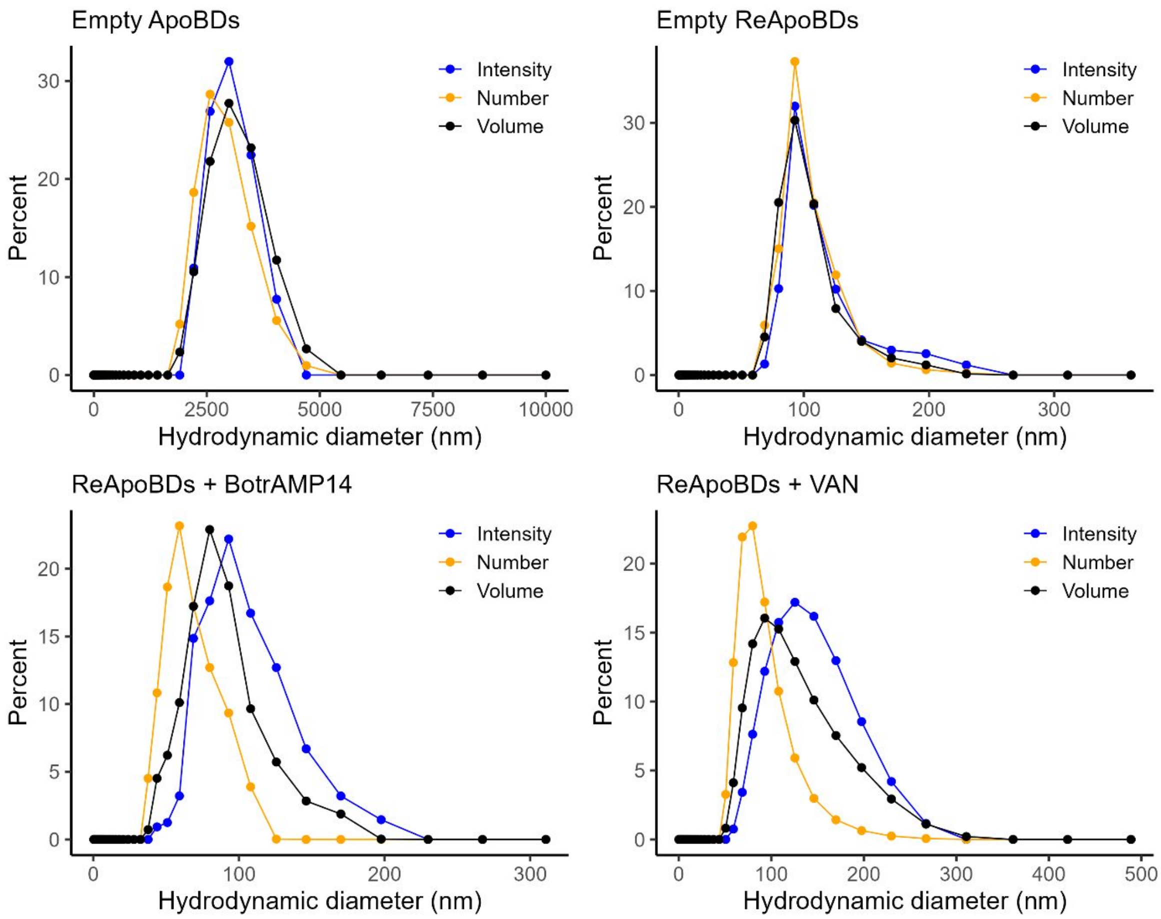

Table. S2.

Minimum inhibitory concentrations (MIC) of free compounds and nanoformulations against *S. aureus* Aurora. Values represent the lowest concentration (μM) that inhibited bacterial growth compared to the untreated control.

Treatment	MIC (μM)	Replicates (n)
Vancomycin (VAN)	0.5	3
ReApoBD+VAN	0.5	3
BotrAMP14	4	3
ReApoBD+BotrAMP14	4	3

***** MIC was defined as the lowest antimicrobial concentration at which no visible growth was observed, in accordance with CLSI-M100 guidelines (2022).This corresponded to OD_{600} values ≤ 0.1 above the blank medium. These OD values reflect ≥ 90 % inhibition relative to untreated control, following approaches used in high-throughput MIC determination.

Fig. S8.

Theoretical response of macrophages to intracellular drug treatments based on cytotoxicity and antimicrobial activity. The figure illustrates how treatment outcomes vary depending on the balance between intracellular cytotoxic effects and intracellular antimicrobial efficacy.

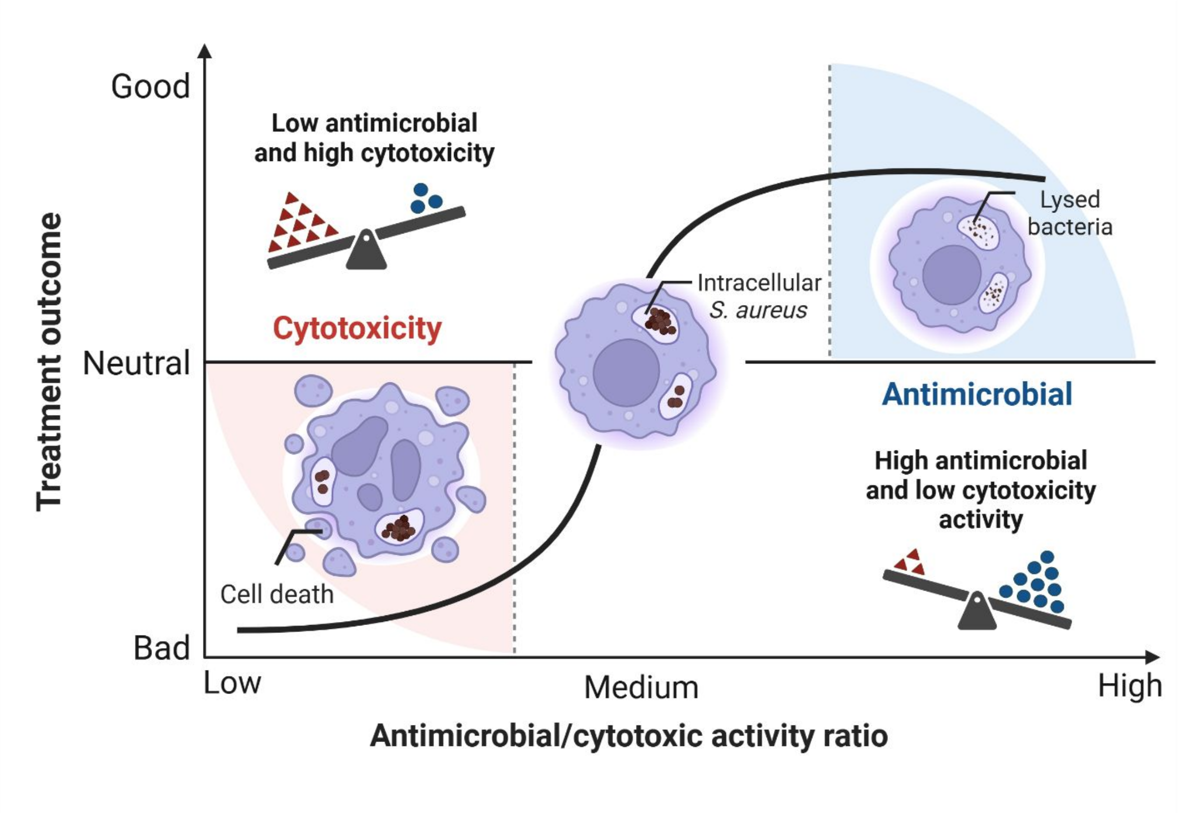

Fig. S9.

Three-dimensional visualization of the intracellular uptake of the antimicrobial peptide and its nanoformulation using confocal microscopy. (**A**). 3D reconstruction of RAW 264.7 cells treated with BotrAMP14-NH2-TAMRA for 20 min with no detectable TAMRA signal, indicating that the non-nanoformulated peptide fails to internalize into the macrophages. (**B**). 3D reconstruction of RAW 264.7 cells treated with ReApoBDs-BotrAMP14-NH2-TAMRA for 60 min. The image highlights successful peptide internalization, as evidenced by a high number of fluorescent vesicles (green and yellow) that are colocalized within the cytoplasm. This analysis underscores the effectiveness of the nanoformulation in promoting cellular uptake.

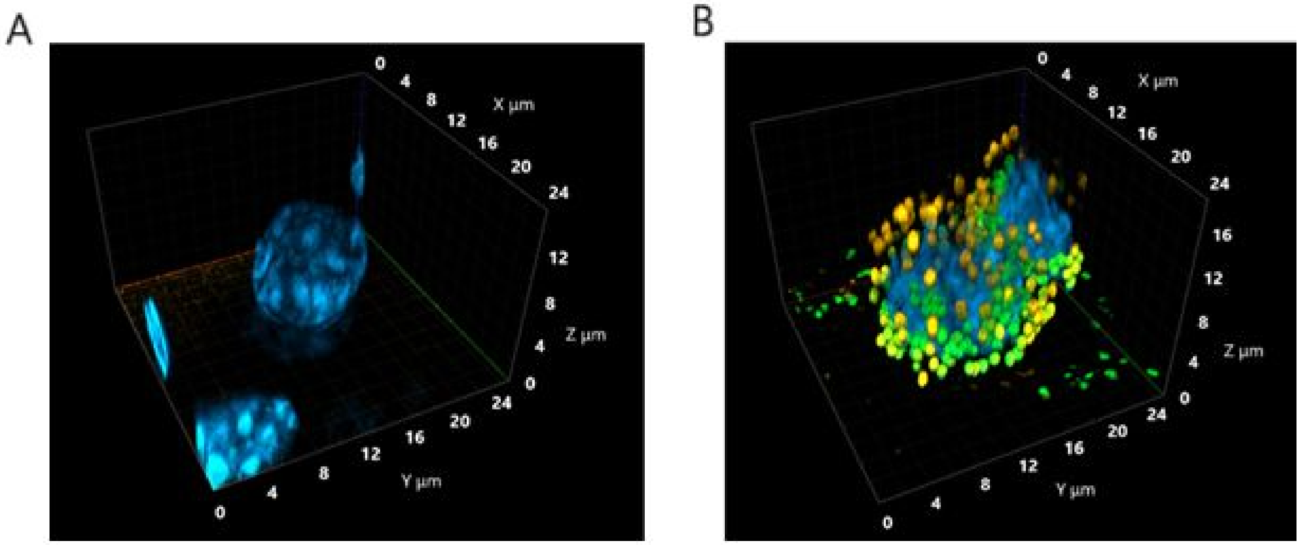

Fig. S10.

Flow chart of the experimental design. The diagram outlines the experimental steps for the production and purification of apoptotic bodies (ApoBDs) from HeLa and BV-2 cells, as well as the production, characterization, and antimicrobial evaluation of a reconstructed BV-2 ApoBD (ReApoBD) nanoformulation with vancomycin (VANH) and BotrAMP14.

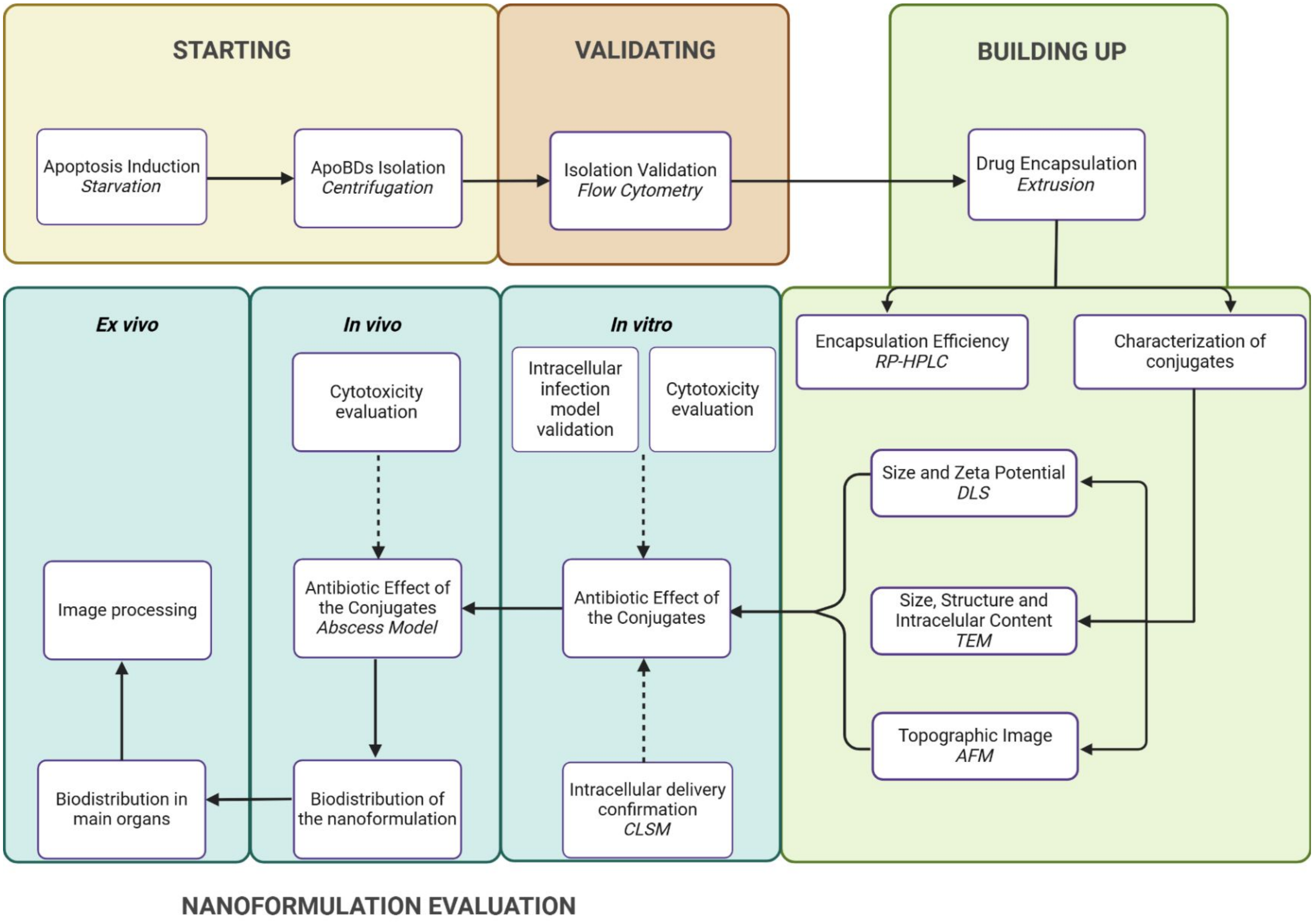

Supplement: Supplementary file 1 [file mt5c01222_si_001.pdf]
